# Sperm activate TLR2/TLR1 heterodimerization to induce a weak proinflammatory response in the bovine uterus

**DOI:** 10.3389/fimmu.2023.1158090

**Published:** 2023-04-27

**Authors:** Alireza Mansouri, Mohamed Samy Yousef, Rasoul Kowsar, Nonoka Usui, Ihshan Akthar, Akio Miyamoto

**Affiliations:** ^1^ Global AgroMedicine Research Center (GAMRC), Obihiro University of Agriculture and Veterinary Medicine, Obihiro, Japan; ^2^ Department of Theriogenology, Faculty of Veterinary Medicine, Assiut University, Assiut, Egypt; ^3^ Department of Animal Sciences, College of Agriculture, Isfahan University of Technology, Isfahan, Iran

**Keywords:** sperm, endometrium, Toll-like receptor 2, dimerization, inflammation

## Abstract

Toll-like receptor 2 (TLR2) signaling pathway is involved in the sperm-triggered uterine inflammatory response at insemination, but its precise mechanism at molecular-level remains unknown. According to the ligand specificity, TLR2 forms a heterodimer with TLR1 or TLR6 as an initial step to mediate intracellular signaling, leading to a specific type of immune response. Hence, the present study aimed to identify the active TLR2 heterodimer (TLR2/1 or TLR2/6) that is involved in sperm-uterine immune crosstalk in bovine using various models. First, *in-vitro* (bovine endometrial epithelial cells, BEECs) and *ex-vivo* (bovine uterine explant) models were employed to test different TLR2 dimerization pathways in endometrial epithelia after exposure to sperm or TLR2 agonists; PAM3 (TLR2/1 agonist), and PAM2 (TLR2/6 agonist). Additionally, *in-silico* approaches were performed to confirm the dimer stability using *de novo* protein structure prediction model for bovine TLRs. The *in-vitro* approach revealed that sperm triggered the mRNA and protein expression of TLR1 and TLR2 but not TLR6 in BEECs. Moreover, this model disclosed that activation of TLR2/6 heterodimer, triggers a much stronger inflammatory response than TLR2/1 and sperm in bovine uterine epithelia. In the *ex*-*vivo* model that mimics the intact uterine tissue at insemination, sperm also induced the protein expression of both TLR1 and TLR2, but not TLR6, in bovine endometrium, particularly in uterine glands. Importantly, PAM3 and sperm induced similar and low mRNA expression of pro-inflammatory cytokines and TNFA protein to a lesser extent than PAM2 in endometrial epithelia. This implied that sperm might trigger a weak inflammatory response *via* TLR2/TLR1 activation which is similar to that of PAM3. Additionally, the *in-silico* analyses showed that the existence of bridging ligands is essential for heterodimer stability in bovine TLR2 with either TLR1 or TLR6. Altogether, the present findings revealed that sperm utilize TLR2/1, but not TLR2/6, heterodimerization to trigger a weak physiological inflammatory response in the bovine uterus. This might be the way to remove excess dead sperm remaining in the uterine lumen without tissue damage for providing an ideal uterine environment for early embryo reception and implantation.

## Introduction

In bovine, during natural breeding or artificial insemination (AI), a massive number of sperm are introduced into the female reproductive tract (FRT) of the estrus animal to increase the probability of fertilization. In the course of the sperm’s journey to the ova, sperm interact with the immune system of FRT.

Different Toll-like receptors (TLRs) have been found to be involved in the induction of inflammatory responses in the FRT (1–7). TLR2 play a key role in binding and immune-cross talk between sperm and FRT (1–3, 5–8). Active sperm cells bind to the bovine endometrium *via* the TLR2 and induce pro-inflammatory responses. However, sperm attachment to bovine oviduct epithelial cells is mediated also by TLR2 but leads to an anti-inflammatory response. The ovum releasing from the ovary lead to a tightly regulated sterile inflammatory response in bovine oviduct which is rapidly resolved during early corpus luteum formation (9). Meanwhile, TLR2 is also expressed in cumulus cells of cumulus-oocyte complexes (COCs) and plays immune protective functions critical for cell survival during ovulation and fertilization (10, 11).

Notably, sperm-induced weak inflammation in bovine endometrium has an essential role in uterine clearance prior to accept the embryo (3, 12). Despite numerous previous studies, the detailed molecular mechanism of sperm-uterine inflammatory signaling that regulated by TLR2 remains unclear.

TLRs are transmembrane proteins, consisting of three main domains: Extracellular domain or leucine-rich repeats (LRR), Transmembrane domain and Intracellular domain or Toll/IL-1R (TIR) domain. Ligand-induced dimerization plays a critical role in signaling through TLRs, in particular TLR2 and TLR4, due to the need for two TLR domains in the vicinity of each other to initiate TLR signaling cascade, consequently recruiting the main TLR domain-containing adaptors: MyD88, MAL/TIRAP, TRIF, TRAM, and SARM (13–15).

Ligand-induced dimerization has been proposed as a key event in the activation of TLR2 in human and mouse models (13, 14). Interestingly, TLR2/1 heterodimer has been more associated with a pro-inflammatory response compared with TLR2/6 complex, which has been shown to be related to both pro- and anti-inflammatory responses (13–16). Of note, TLR2 is involved in pro-inflammatory responses in the bovine uterus against sperm (1–3, 7).

Since dimerization is a prerequisite for any TLR2 activation, we hypothesized that the sperm-triggered physiological inflammatory response in bovine endometrium is regulated by different types of TLR2 dimerization (TLR2/1 or TLR2/6), though the structure of TLR2 dimers in bovine is unknown. *In-vitro* cell cultures using BEECs are considered as the starting point for studying any biological effect in bovine uterus (2, 7). However, this model cannot simulate the anatomical complexity of bovine uterus especially for sperm-uterine interaction (particularly uterine gland). Thus, the *ex-vivo* model using uterine explant has been performed to mimic the *in-vivo* conditions to identify different physiological interactions in bovine uterus (1).

In this study, we first employed *in-vitro* approach to evaluate the sperm-triggered TLR1, 2 and 6 expressions in endometrial epithelial cells to determine which TLR2 heterodimer is activated by sperm in bovine endometrium. Then using the same model, the degree of inflammatory response by different TLR2 agonists (i.e., PAM3 and PAM2) was evaluated and compared to sperm induced inflammation. Thereon, *ex-vivo* uterine explant culture model was used as a powerful and more physiological tool to verify the possible TLR2 dimerization mechanism in response to sperm in the uterine tissue in particular in the uterine glands.

Protein–protein interactions (PPI), including dimerization and protein complex, control all functions of the living cell during physiological and pathological conditions (17, 18). Recently, different *in-silico* approaches have been used to identify the biological pathways of PPI and highlight possible applications (19–21). TLRs molecular-level responses are extensively studied using computational biology approaches (19–21). Hence, *in-silico* model was employed for the first time to investigate dimerization process of TLR2 at molecular-level in bovine model. At first, the binding affinity of TLR2 with TLR1 and TLR6 was evaluated using known crystal structures of both mouse and human. Afterwards, the potential effect of PAM3 and PAM2 (TLR2/1 and TLR2/6 agonists, respectively) on TLR2 dimerization was investigated *via de novo* generated bovine TLRs.

## Methodology

### Study design

**Figure 1:**
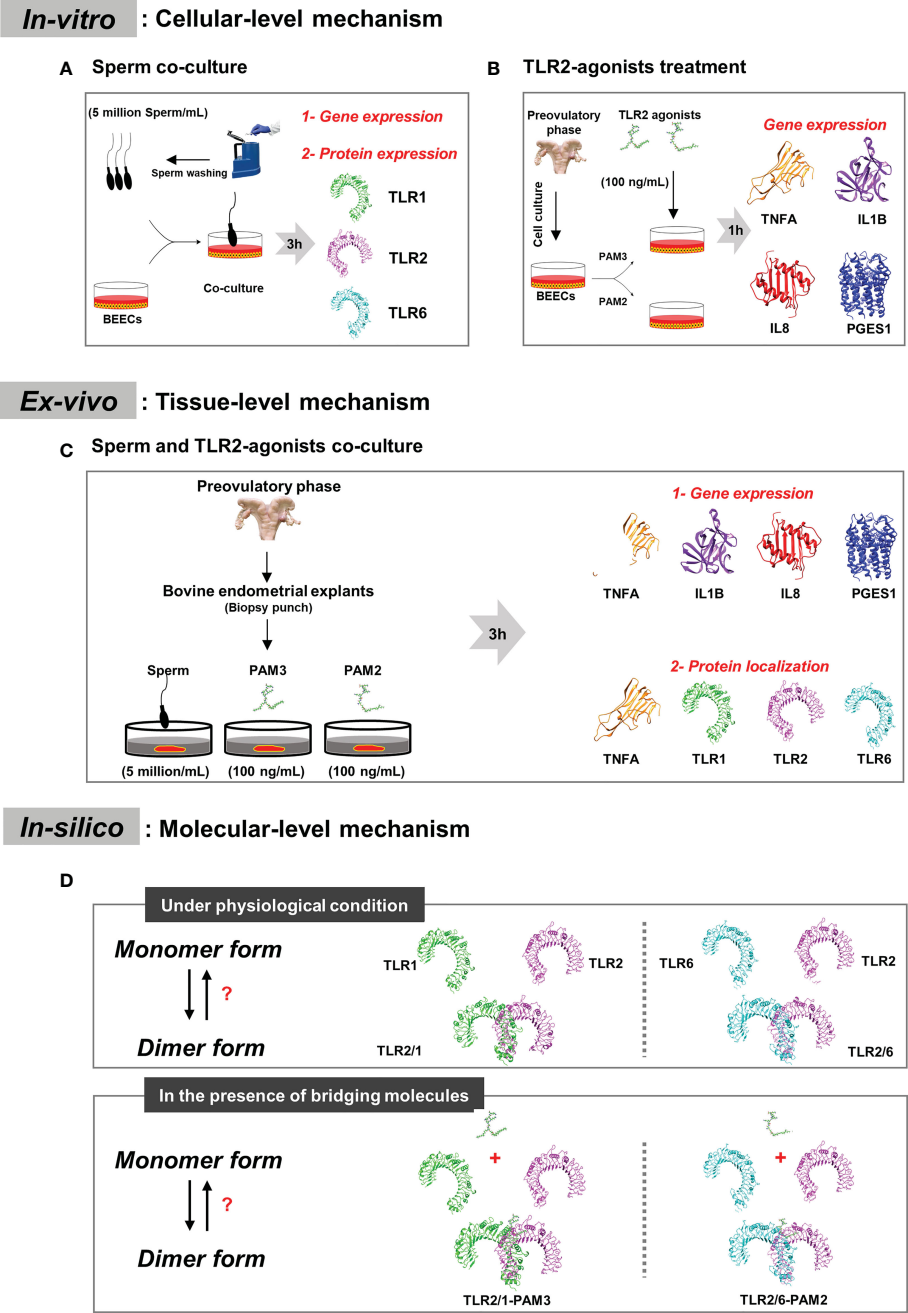
Plan representation of the research design. **(A)** *In-vitro* model: to detect which active TLR2 dimer can be employed by sperm in bovine endometrium at cellular level. BEECs monolayer were co-cultured with 5million sperm per mL for 3h. qRT-PCR and immunostaining analyses were done to investigate TLRs mRNA and protein expression. **(B)** *In-vitro* model: to study inflammation intensity through the two classical TLR2 signaling (TLR2/1 and TLR2/6 signaling cascades), BEECs monolayer were stimulated with 100 ng/mL of PAM3 (TLR2/1 agonist) and PAM2 (TLR2/6 agonist) for 1h. A time-dependent experiment (1, 6 and 12h) was done to confirm the difference between both TLR2 signaling pathway. Finally, qRT-PCR analysis was performed to consider the pro-inflammatory mRNA expression (*TNFA*, *IL1B*, *IL8* and *PGES1*). **(C)** *Ex-vivo* model: to compare sperm induced inflammation in endometrium with PAM3 and PAM2. Bovine endometrial explants were co-incubated with 5milion sperm/mL, and 100 ng/mL PAMs for 3h. First, qRT-PCR was employed to investigate the pro-inflammatory mRNA expression. Afterwards, immunostaining was used to evaluate and localize the strong inflammatory marker (TNFA) for all groups and signaling marker proteins (TLR1, TLR2 and TLR6) in endometrium for sperm group. **(D)** *In-silico* model: we investigated dimerization process for TLR2 in different condition, in absent and presence of TLR2 agonists. Additionally, we predict the effect of agonist on bovine TLR dimerization.

*In-vitro*, *ex-vivo* and *in-silico* investigations were conducted to define the signaling mechanism by which TLR2 is regulated during sperm-uterine immune interactions in non-pregnant cattle (**Figure 1**).

#### *In-vitro* approaches

##### Experimental design and *in-vitro* studies

In order to elucidate the heterodimeric form of TLR2 signaling in sperm- induced inflammation in bovine uterus at cellular level, BEECs were co-cultured with sperm (5 million/mL) for 3h. Furthermore, to investigate the contribution of TLR2 signaling (TLR2/1 and TLR2/6) cascade to uterine inflammation, the following experiments was conducted using different TLR2 agonists. Bovine endometrial epithelial cells (BEECs) were stimulated with PAM3 (TLR2/1 agonist, ab142085, Abcam) and PAM2 (TLR2/6 agonist, InvivoGen, USA) at 100 ng/mL concentration for 1h. The concentration of dose and time point were selected based on our previous reports in which those conditions were investigated in detail (2, 7). In brief, based on dose- and time-dependent investigations (10^4^, 10^5^ and 10^6^ sperm/mL), 5 million/mL of sperm was used to induce the weak physiological inflammation after co-culture with BEECs for 3 h (8). As well, PAM3 (10, 100 and 1000 ng/mL) was applied, and 100 ng/ml was the first to induce the inflammatory response in BEECs at 1h of incubation in the similar level to that of sperm (2). Thus, 100 ng/mL of PAM3 and the same concentration of PAM2, were used in the present study to compare their inflammatory effects with sperm.

##### BEECs Culture

Initially, macroscopically healthy non-pregnant bovine uteri were carefully observed to be free of inflammation and abnormal color or any pathological lesions in slaughterhouse (Obihiro, Hokkaido, Japan), then collected and directly transferred to the laboratory under sterilized conditions and the uterine horn was used to isolate epithelial cells (2, 7). The isolated cells were cultured in Dulbecco’s Modified Eagle Medium: Nutrient Mixture F-12 (DMEM/F12, Gibco, Grand Island, USA) supplemented with 1% amphotericin B, 0.1% gentamicin (Sigma-Aldrich, Steinheim, Germany), 10% heat-inactivated fetal calf serum (FCS) (Biowest USA) and 2.2% NaHCO3 using flask. The culture medium was replaced regularly with new media every 48 h. Upon reaching 70–80% confluence, the cells were collected with trypsinizing (0.05% trypsin EDTA; Amresco, Solon, OH, USA), transferred in 24-well and 12-well plates (Nalge Nunc International, Roskilde, Denmark) and cultured up to around 90% confluence (first passage). Estrogen (E2) and progesterone (P4) were added at preovulatory concentrations in the cell culture media (DMEM/F12, 1% amphotericin B, 0.1% gentamicin and 5% FCS) (2, 7).

##### BEECs co-cultured with sperm

The sub-confluent BEEC monolayers (after first passage) were washed twice with PBS and cultured in a medium supplemented by 0.1% FCS and gentamicin. The BEECs were co-cultured with 5 million/mL washed sperm, followed by washing cell twice with PBS, lysing with Trizol (Invitrogen, Carlsbad, USA), and storing at −80°C until RNA extraction. This experiment was repeated seven times using epithelial cells from seven different uteri (n=7). For preparing washed sperm, frozen semen straws (obtained from three Holstein bulls kept in the Genetics Hokkaido Association, Hokkaido, Japan) were thawed at 38.5°C for 30 sec, followed by washing three times at 200g for 10 min using sp-TALP (2, 7, 8). The sp-TALP consisted of 99 mM NaCl, 3.1 mM KCl, 25 mM NaHCO_3_, 0.39 mM NaH_2_PO_4_, 10 mM HEPES free acid, 2 mM CaCl_2_, 1.1 mM MgCl_2_, 25.4 mM sodium lactate, 0.11 mg/ml sodium pyruvate, 50 µg/ml gentamycin and 6 mg/ml BSA (Sigma-Aldrich, USA) pH 7.4.

##### Stimulation of BEECs with agonists

The sub-confluent BEEC monolayers (after first passage) were washed twice with Phosphate Buffered Saline (PBS) and cultured in a medium supplemented by 0.1% FCS and gentamicin. The BEECs were either stimulated by 100 ng/mL PAM3 and PAM2 for 1, 6, 12 h. At the end of BEECs stimulation, cells were washed twice with PBS, lysed with Trizol (Invitrogen, Carlsbad, USA), and stored at −80°C until RNA extraction. This experiment was repeated seven times using epithelial cells from seven different uteri (n=7).

#### *Ex-vivo* approaches

##### Experimental design

In order to compare the endometrial response toward sperm and TLR2 agonists at the preovulatory phase, an *ex-vivo* model (bovine endometrial explants) was used, due to the advantage of investigating the protein localization in different compartments of the endometrium and the links between whole-animal condition and cellular function.

##### Sperm and agonist co-incubation with endometrial explants

Bovine endometrial explants were prepared as described previously (1). Briefly, pre-ovulatory bovine uteri were observed to be free of inflammation and abnormal color or any pathological lesions in slaughterhouse (Obihiro, Hokkaido, Japan), then collected and directly transferred to the laboratory for *ex-vivo* investigations under sterilized conditions. Afterwards, using an 8 mm biopsy punch, endometrial explant tissue disks were extracted from the glandular (intercaruncular) endometrial regions. Next, explants disks were placed into a plate with sp-TALP and put in the incubator (38.5°C and 5% CO_2_) for 15 min (1).

To compare the sperm induced inflammation with agonists, explants were incubated with sperm (5 million sperm/mL) and TLR2 agonists (100 ng/mL) for 3h in the incubator (38.5 ^0^C, 5% CO_2_, 0.5mL sp-TALP per well in 24-well plate) and at the end of the incubation period the explants were processed for RNA extraction and immunofluorescence analysis. This experiment was repeated five times using explants from five different uteri (n=5).

##### PCR protocol

BEECs and uterine explants were subjected to RNA extraction, cDNA synthesis, and quantitative real-time PCR were done through following previous protocol (8). Trizol reagent (Thermo Fisher Scientific) were used to extract total RNA, followed by measuring RNA concentration using a spectrophotometer (Eppendorf, Munich, Germany), after that stored in RNA storage solution (Ambion, Austin, TX, USA) at −80°C until cDNA conversion step. The cDNA synthesis was performed as previously described (8), and the synthesized cDNA was stored at −30°C. Quantitative real-time PCR of target genes (*TNFA, IL1B, TLR2, TLR1, TLR6, IL8, PGES1* and *β-actin*, **Supplementary Table 1**) was carried out by QuantiTect SYBR Green PCR Master Mix (QIAGEN GmbH, Hilden, Germany) using an iCycler iQ (Bio-Rad Laboratories, Tokyo, Japan) (8). The calculated cycle threshold values were normalized using *β-actin* as an internal housekeeping gene by applying the Delta-Delta comparative threshold method to quantify the fold change between samples.

##### Immunofluorescence protocol

a) IF for monolayer cells

At first, monolayer cell was cultured on 24-well plates with 13mm diameter glass coverslips and grow to 90% confluence then co-cultured with 5 million sperm per mL for 3h. Cells were washed with PBS twice and fixed with 2 mL of 4% formaldehyde for 15 min at RT, followed by washing twice with PBS. After that, the cells were permeabilized with 2 mL of 0.1% Triton-X10 in PBS for 15 min on ice, followed by washing three times with PBS. Afterwards, the monolayer cells were blocked using 2mL blocking buffer (5% BSA in PBS) for 1h at RT. The cells were incubated with primary antibodies for TLR1, TLR2 and TLR6 (**Supplementary Table 2**) in humid chamber at 4°C overnight. After washing five times with PBS, the cells were incubated with Alexa Flour conjugated secondary antibody (**Supplementary Table 2**) for 1h at 4°C. After that, the cells were washed six times with PBS, followed by mounting in VECTASHIELD mounting medium containing DAPI (H-1200, Vector Laboratories, CA, USA).

b) IF for explant tissue

After the incubation, explants were rinsed in sp-TALP and fixed in 4% paraformaldehyde solution. Then, the fixed tissue samples were dehydrated using ethanol gradient (70, 80, 90, 95 and 100%), cleared in absolute alcohol and xylene, followed by embedding in paraffin and sectioning in 5 μm thick slices. The endometrial sections were deparaffinized and rehydrated through placing on xylene, absolute alcohol and grades series of alcohol, in turn. After that, the tissue sections were blocked with normal goat serum (1:50, S-1000, Vector Laboratories, CA, USA) for 30 min at RT and followed by incubating overnight with primary antibodies for TLR1, TLR2, TLR6 and TNFA (**Supplementary Table 2**) at 4°C in a humidified chamber. Afterwards, the sections were incubated with Alexa Fluor conjugated secondary antibodies (**Supplementary Table 2**) for 30 min. Sections were washed, and coverslips were mounted using VECTASHIELD mounting medium containing DAPI. Finally, the sections were observed under fluorescence microscope (BZ-X800, Keyence).

#### *In-silico* approaches

##### Preparation of the molecules

In order to investigate TLR2 dimerization under both physiological state and agonist stimulation in a bovine model, an *in-silico* approach was conducted. To aim that, the TLR2/1-PAM3 (PDB ID: 2Z7X) and TLR2/6-PAM2 (PDB ID: 3A79) were selected for this research. The crystal structures of the TLRs extracellular domain of human (*Homo sapiens*) and mouse (*Mus musculus*) species are known (PDB ID of 2Z7X and 3A79, respectively) but have not yet been crystallized in bovine species (*Bos taurus*). Hence, we carried out Basic Local Alignment Search Tool (BLAST) to calculate the local similarity between bovine TLRs with human and mouse TLRs. It revealed an identity of 77.6% (Human - Bovine TLR2), 78.5% (Human - Bovine TLR1), 66.7% (Mouse - Bovine TLR2) and 72.1% (Mouse - Bovine TLR6).

##### Investigating the affinity between TLRs in heterodimer forms

The crystal structure of TLRs in the presence and absence of agonist, and the obtained initial structure from Haddock online server were used to investigate the affinity between TLRs (three repeats). For applying TLRs to the Haddock 2.4 web server (22), the residues involved in h-bond in TLRs interaction obtained through Ligplot analysis (23) were selected as active residues at the contacting site of TLRs. To obtain the initial structure for MD simulation, cluster 1 was selected as the best docked complex based on the highest HADDOCK score (according to the following formula: Score: 1.0 * Evdw + 0.2 * Eelec + 1.0 * Edesol + 0.1 * Eair). To calculate the binding free energies between TLRs in heterodimer forms, 150 ns MD simulation were applied to obtain trajectory MD simulation, followed by calculating binding free energy using molecular mechanics/Poisson–Boltzmann surface area (MM/PBSA) method (24–32) (**Supplementary Data**; **Supplementary Figures 1–4**).

##### The prediction of binding pockets of TLRs in human, mouse and bovine

The possible binding pockets on TLRs were identified through DoGSite Scorer web server, which is a strong tool for investigating potential binding pockets (33, 34). The DoGSite Scorer web server is used for mapping the possible binding pockets based on descriptors calculation (such as depth (A), surface (A^2^), volume (A^3^)). Furthermore, the druggability score is estimated through the support vector machine (SVM) method. The score of druggability classified from 0 to 1 while higher values are the potential pockets for the main binding sites. The pocket detection and analysis were performed for TLRs of three mammalian species (crystal structures of human, mouse, and *de novo* modeling of Bovine TLRs). Bovine TLR protein 3-D structure prediction was carried out after applying amino acid sequences of extracellular domain (Uniprot code: Q95LA9, B5TYW4 and Q704V6 for TLR2, TLR1 and TLR6, respectively) to I-TASSER server (27), followed by optimizing 3-D structures using 100 ns MD simulation.

### Statistical analysis

The statistical analysis was conducted with SPSS*^®^* software version 22 (IBM, Armonk, Ny, USA). The data were first tested for normality using Kolmogorov–Smirnov test. A non-parametric Kruskal–Wallis test followed by a Mann–Whitney test were applied for non-normally distributed data of mRNA gene expressions. While One-way analysis of variance (ANOVA) and *post hoc* Tukey’s test were used for normally distributed data obtained from TNFA immunofluorescence analysis. An unpaired two-tailed parametric Student’s t-test was performed to evaluate the differences between two unpaired groups. The statistical significance was defined as P< 0.05.

## Result

### TLR2/1 heterodimer employed by sperm to induce inflammation in BEECs

**Figure 2:**
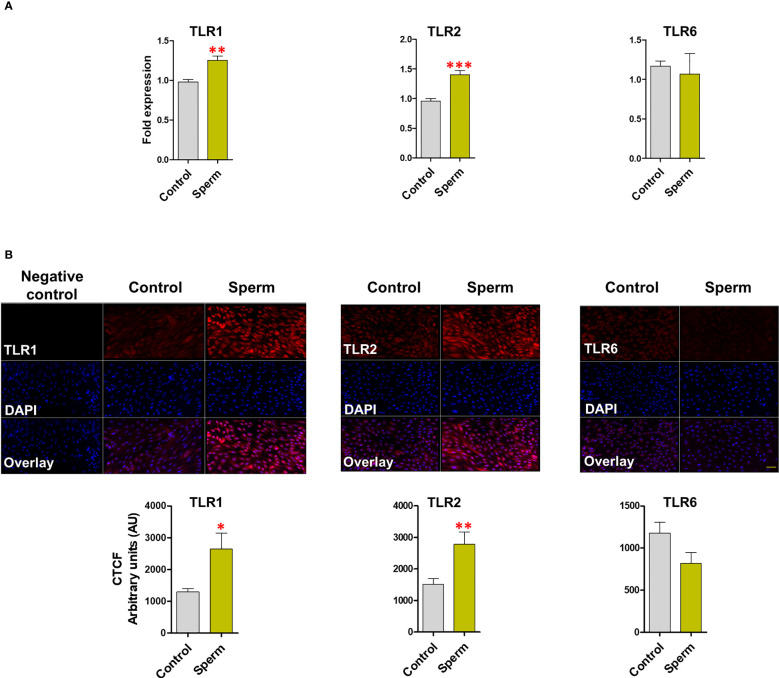
Sperm induce TLR2 and TLR1 (not TLR6) expression in BEECs. **(A)** Relative mRNA expression of *TLR1, 2* and *6* in BEECs after 3h co-culture with 5 million/mL sperm. **(B)** Immunofluorescence staining of TLR1, 2 and 6 in BEECs monolayer. This experiment was repeated seven times using epithelial cells from seven different uteri. Asterisks show a significance of difference (*P < 0.05, **P < 0.01, or ***P < 0.001) in the treatment group when compared to the control group. Bar = 50 µm.

In BEECs, sperm induced *TLR2* (P <0.001) and *TLR1* (P <0.01) mRNA expression, but not *TLR6* (**Figure 2A**). Moreover, the immunofluorescence analysis showed similar expression profiles for TLR2 (P <0.01) and TLR1 (P <0.05) after sperm co-culture with BEECs (**Figure 2B**).

### The varying degrees of inflammatory reaction following endometrial activation of TLR2/1 and TLR2/6

**Figure 3:**
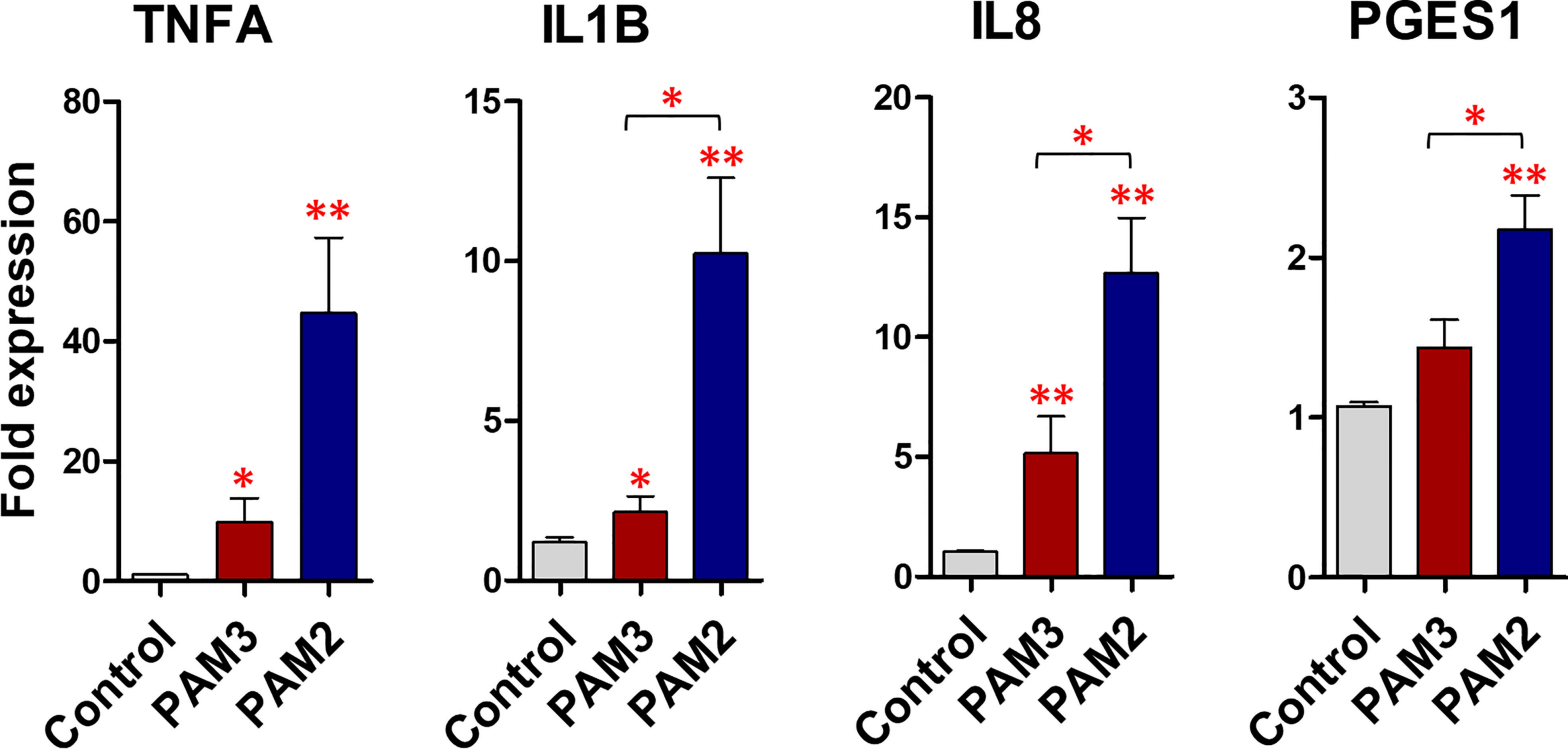
PAM3 and PAM2 induce a weak and strong inflammatory response, respectively, in BEECs. The relative mRNA expression of pro-inflammatory cytokines and *PGES1*, after stimulation with 100 ng/mL PAM2 and PAM3 for 1h. This experiment was repeated seven times using epithelial cells from seven different uteri. Asterisks show a significance of difference (*P < 0.05 or **P < 0.01, Mann–Whitney test).

The PAM3 increased the mRNA expression of *TNFA* and *IL8* (P <0.05) compared to the control. Meanwhile PAM2 significantly increased the mRNA expression of *TNFA* (P <0.01), *IL1B* (P <0.01), *IL8* (P <0.001), and *PGES1* (P <0.001) compared to the control. Compared to PAM3, PAM2 treatment significantly (P <0.05) increased the transcription levels of the pro-inflammatory genes (*TNFA*, *IL1B*, *IL8*, and *PGES1*). For instance, *TNFA*, *IL1B*, and *IL8* expressions were roughly 3-fold higher in the PAM2 group than in the PAM3 treatment (**Figure 3**). Additionally, a time-dependent exposure revealed that PAM2 over PAM3 increased *TNFA* mRNA expression in BEECs at various time points. (1, 6 and 12h) (**Supplementary Figure 5**).

### Sperm induced the TLR2/1 protein expression in endometrial explants

**Figure 4:**
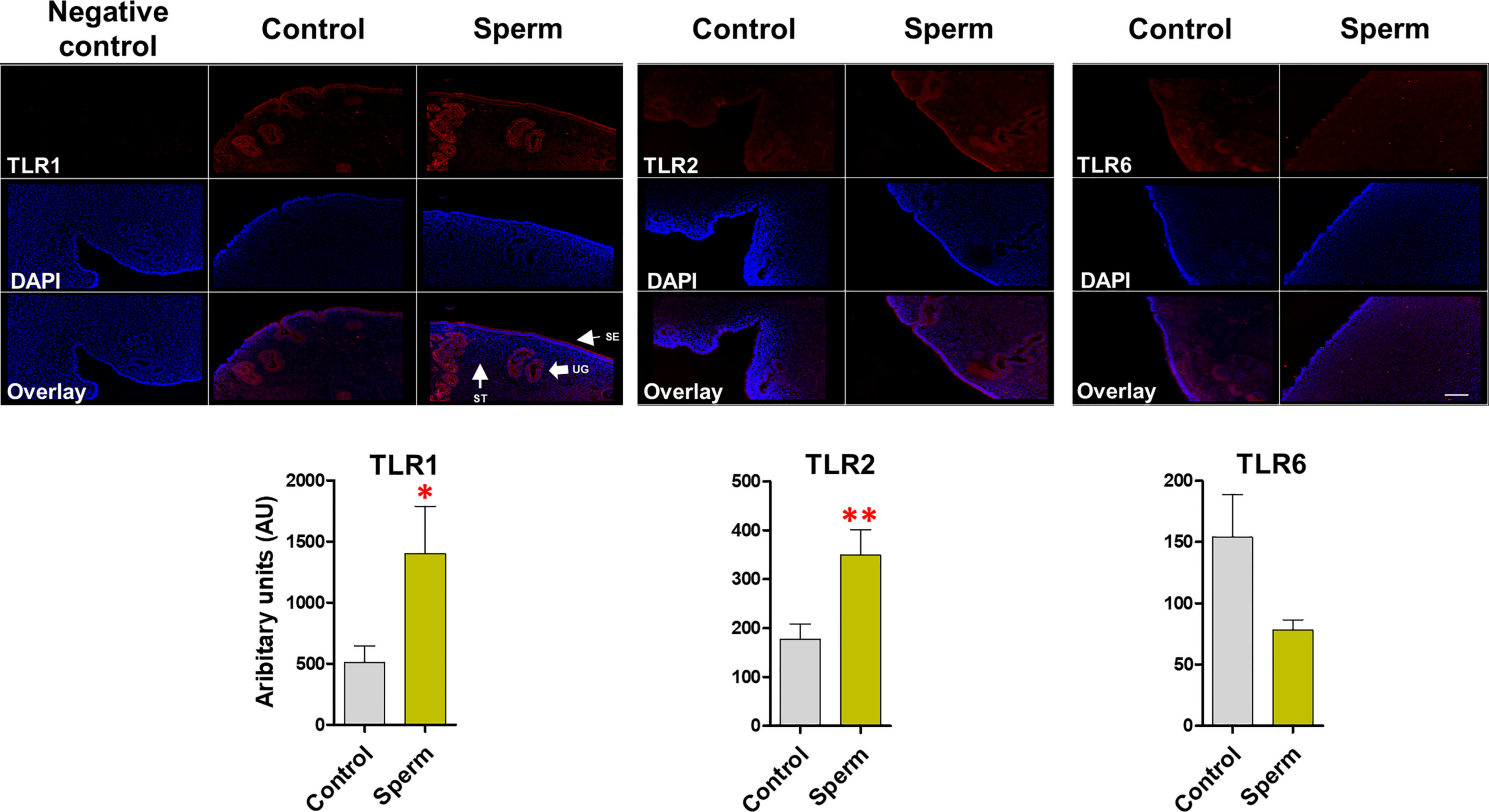
Sperm induce TLR2 and TLR1 (not TLR6) protein expression in bovine endometrial explants. The intense expression of TLR1 and TLR2 in uterine glands (UGs) and surface epithelium (SE) in sperm group. This experiment was repeated three times using explants from three different uteri. Asterisks show a significance of difference (*P < 0.05 or **P < 0.01) in the treatment group when compared to the control group. when compared to the control group. ST, stroma. Bar = 100 µm.

The immunofluorescence analysis revealed that, TLR1, TLR2 and TLR6 is localized in the bovine endometrium, particularly in surface and glandular epithelium. It was obvious that sperm induce the protein expression of TLR2 (P <0.01) alongside TLR1 (P <0.05) in similar manner, in particular in the uterine gland and in the surface epithelium. On the other hand, for TLR6 expression, the intensity was not modulated compared to the control in the uterine gland and surface epithelium after sperm interaction with endometrial epithelia (**Figure 4**).

### Sperm-induced inflammation in bovine endometrium is similar to that of PAM3 (TLR2/1 pathway)

**Figure 5:**
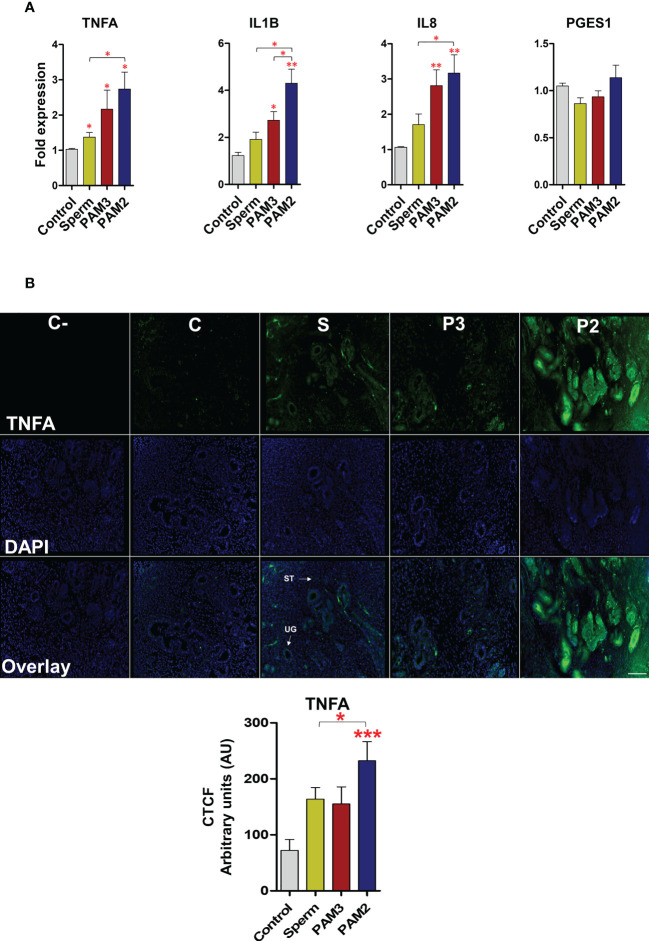
Sperm and PAM3 induce a weaker inflammation in bovine endometrial explants, compared with PAM2. **(A)** Relative mRNA expression of pro-inflammatory cytokines and *PGES1* after stimulation with 5 million/mL sperm, 100 ng/mL PAM3 and PAM2 for 3h. This experiment was repeated seven times using epithelial cells from seven different uteri. **(B)** Immunofluorescence staining for TNFA in endometrial explants **(C**: Negative control, C: Control, S: Sperm, P3: PAM3 and P2: PAM2). This experiment was repeated three times using explants from three different uteri. Asterisks show a significance of difference (*P < 0.05, **P < 0.01 or ***P < 0.001 , Mann–Whitney test) between the means of each two independent groups. Bar = 100 µm.

PAM2 induced a higher (P < 0.05) mRNA expression of pro-inflammatory cytokines (*TNFA*, *IL1B and IL8*) in uterine explant compared to the control and sperm treatments. Of note, there was no significant difference (P > 0.05) between sperm and PAM3 groups for mRNA expression of the investigated cytokines (**Figure 5A**). However, *PGES1* gene expression did not show a significant change after stimulating the uterine explant with PAM3, PAM2 or sperm. In the same way, the intensity of TNFA protein expression was highly significant after PAM2 treatment in comparing to sperm and control (P < 0.05) particular in uterine gland of the endometrium compared to the control (**Figure 5B**).

### Identical TLR2 dimerization process in human, mouse and bovine

In the present study, the *in-silico* analysis confirmed that TLR2 is not able to interact with TLR1 at cellular level in human and mouse models (**Supplementary Results**; **Supplementary Table 3**; **Supplementary Figure 6**). In contrast, TLR6 has a low affinity with TLR2. Our *in-silico* analyses clearly indicate that the affinity between TLRs is not considerable to stabilize dimer forms. Thus, the bridging molecules (ligands) are vastly required to stabilize TLR2 dimers (**Supplementary Table 4**).

**Figure 6:**
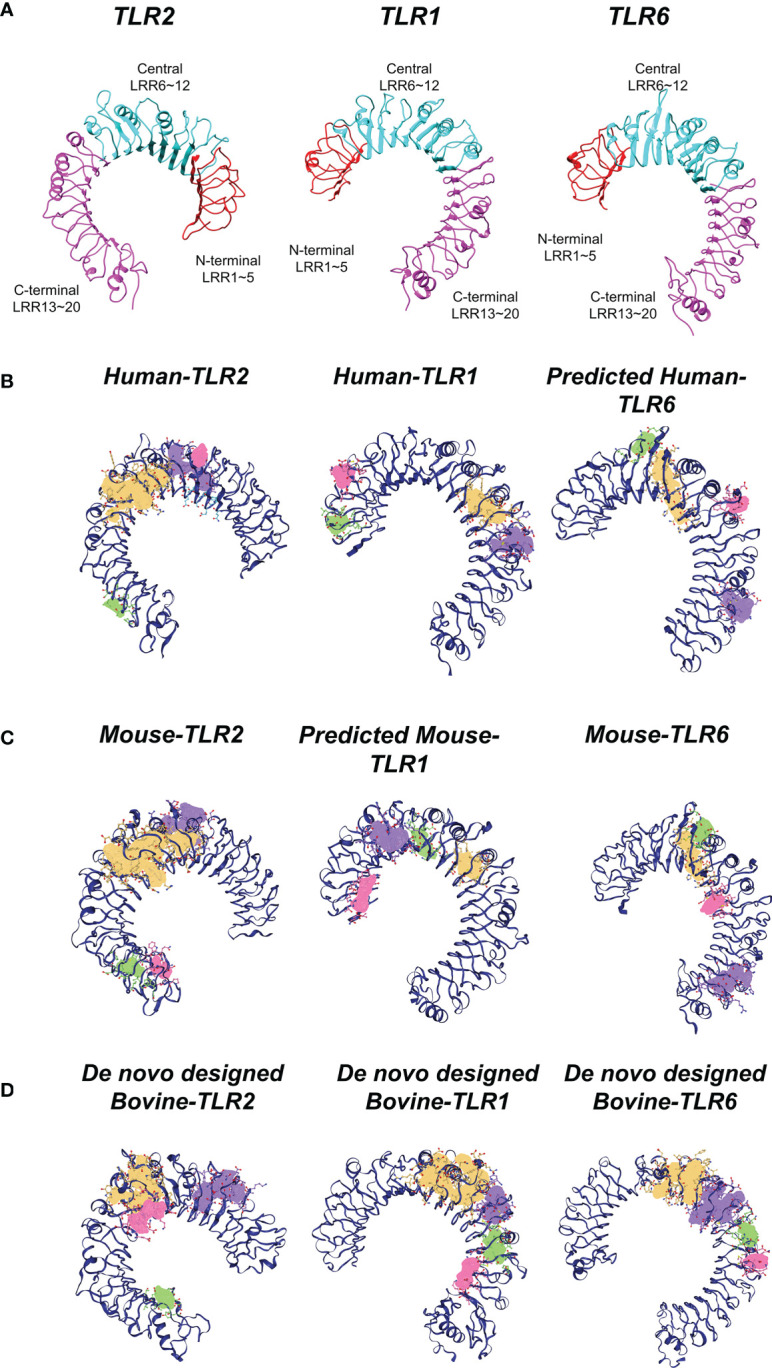
Structural analysis of TLRs in three mammalian classes (human, mouse, bovine). **(A)** Three subdomains of TLRs having 20 LRR units, namely N-terminal (LRRNT), central and C-terminal (LRRCT) in the ectodomain of TLRs. LRRNT (LRR 1~5), central (LRR 6~12) and LRRCT (LRR 13~20) colored by red, cyan and magenta, respectively. **(B)** Predicted potential binding pockets of human TLRs. **C)** Predicted potential binding pockets of mouse TLRs. **(D)** Predicted potential binding pockets of bovine TLRs. Different potential binding pockets of TLRs are shown by yellow (first pocket), violet (second pocket), green (third pocket), red (fourth pocket). The main binding site (yellow pocket) of TLR2 is similar (localized between LRR9-12, and the entrance is in LRR11-12) in the three species. Concerning TLR1, the main binding site (yellow pocket) is between central and LRRCT domains for all species. However, this internal channel is blocked by two phenalene in TLR6.

The ectodomain of TLRs are split into three subdomains: N-terminal (LRRNT), central and C-terminal (LRRCT) (**Figure 6A**). The DoGSite Scorer predicted several binding pockets for all TLRs in humans, mouse and bovine (**Figures 6B–D**). The volume, surface and drugScore of the first predicted binding pockets (yellow pocket) of the all TLR proteins in the three species were indicated in **Supplementary Table 5**. The druggability score for this pocket in all TLRs is >0.7. Moreover, the physicochemical descriptors showed that TLRs of different species were almost identical (**Figure 6**). Based on phyciso–chemical properties, the volume (V) of internal pocket was 1803 Å^3^ (LRR9-12), 1490 Å^3^ (LRR7-12) and 1418 Å^3^ (LRR9-12) in human, mouse and bovine TLR2, respectively. Predicting the main binding pocket for TLR1 in the three species showed that the main binding pockets were located between LRR10-12 for human (V~533 Å^3^) and LRR11-12 for both mouse (V~370 Å^3^) and bovine (V~500 Å^3^). The first predicted binding site of bovine TLR1 was almost identical to human and mouse. However, the volume and the surface were slightly different between species for TLR6 in bovine compared with TLR6 in human and mouse (**Supplementary Table 5**). **Supplementary Figure 7** demonstrated that in the main binding site of TLR2 in three species, the type and sequence of amino acids were identical and conserved (in particular, the residue in the entrance of binding pocket).

Looking at the details of the present data, Phe 322 and Phe 349 in three species were selected as the entrance of binding pockets. Additionally, the data obtained from Ligplot analysis indicated that these Phe residues (349 and 322) play an important role in TLRs interaction with agonist and dimerization process (**Supplementary Figures 6**, **8**). As for TLR1, the predicted main binding pocket for all three species was identical (approximately between Central LRR and c-terminal LRR). With regards to TLR6, in mouse experimentally crystal structure, the Phe 343 and Phe 365 block the internal channel of TLR6, compared with TLR1, consequently, this channel cannot be recognized as main binding site. This structural analysis supposed that the main binding sites of TLRs were similar and located in same place for the studied mammalian species (**Figure 6**).

## Discussion

In this study, using a combination of experimental and *in-silico* modeling, we were able to demonstrate that the sperm-induced inflammatory response activates TLR2/1 heterodimer, but not TLR2/6 in bovine endometrium. Importantly, we revealed that sperm could induce a weak inflammatory response in bovine endometrium through ‘‘PAM3-like-weaker’’ TLR2/1 signalling rather than “PAM2-like-stronger’’ TLR2/6 pathway.

In fact, the TLR2 has an essential role in balancing pro- and anti-inflammatory immune responses in different cell types (2, 10, 16, 35–37). We previously reported that sperm induce transient and weak inflammatory response *via* the regulation of TLR2 signaling in bovine endometrium. On the other hand, heterodimerization of TLR2 with TLR1 or TLR6 has been extensively studied to develop a deep understanding of different immune responses resulted from TLR2 activation. With selective TLR2 heterodimerization, it was important to determine the active TLR2 heterodimer which is involved in sperm-induced inflammation in bovine uterus. Thus, in this study, at first, *in-vitro* BEECs co-culture was employed to assess the expression of TLR1, TLR2 and TLR6 by sperm. Notably, sperm upregulated the mRNA and protein expression of TLR1 and TLR2, but not TLR6 in BEECs, suggesting that sperm utilize TLR2/1 during sperm-uterine interactions. Moreover, the present data showed that the activation of TLR2/6 signaling pathway could lead to a stronger inflammation compared to TLR2/1 in uterine epithelial monolayer using specific agonists. Similarly, Murgueitio et al. (38) indicated that activating TLR2/6 signaling pathway resulted in 4-fold stronger inflammation than TLR2/1 in human embryonic kidney cells. To get the same level of inflammation in this kind of cells, they used 50 ng/mL of PAM2 versus 200 ng/mL PAM3 for 5h (38). In our *in-vitro* model, identical concentration from both PAMs (100 ng/mL) was applied to induce inflammation in bovine endometrial epithelia and interestingly, PAM2 induced a 3-fold higher inflammatory response compared to PAM3 and sperm. Altogether, *in-vitro* studies provided the initial evidence that sperm employ TLR2/1 signaling and activation of TLR2/1 signaling pathway led to a weak inflammatory response in BEECs.

Further, *ex-vivo* experimental model (i.e., preovulatory endometrial explants that physiologically mimic the intact uterine condition at insemination) was used to investigate the localization of TLR1, 2 and 6 in endometrium and to compare the inflammatory intensities of sperm and TLR2 agonists. We previously reported that sperm interact with the uterine glands to induce an acute and weak inflammatory response. This inflammatory response has been detected by the upregulation of key inflammatory markers TNFA, IL1B, IL8 and PGES as well as by the recruitment of inflammatory cells (i.e., neutrophils) (1–3, 7, 12). Notably, in cattle, sperm trigger the inflammatory cascade primarily *via* the TLR2 signaling of uterine glands (1). The present *ex-vivo* protein expression data showed similar upregulation profiles for TLR2 and TLR1, after sperm co-culture with uterine explants. Even though, TLR1, TLR2 and TLR6 localized in surface epithelium and uterine glands, sperm enhanced the expression of TLR1 and TLR2 but not TLR6 in these compartments, which strongly support a co-regulation of TLR2 and TLR1 receptors in particular in uterine glands during sperm-induced inflammatory response. Herein, TLR6 was not modulated after sperm exposure, possibly to prevent strong and long-term inflammatory reactions and tissue damage as high TLR6 that may correlate with higher-risk disease (39). Prominently, the mRNA and TNFA protein profiles of *ex-vivo* model clearly showed that sperm trigger a weak physiological inflammatory response in endometrial epithelia similar to that of the specific ligand of TLR2/1 heterodimer (PAM3). TNFA is one of the key inflammatory markers in the bovine uterus towards sperm in both physiological and pathological conditions (1, 7). Meanwhile, activation of TLR2/6 heterodimers led to a stronger inflammatory response in endometrial explants which assumed to be far from physiological sperm-triggered inflammation. Thus, these *ex-vivo* evidence reveal that, sperm employ TLR2/1 heterodimerization, in particular in uterine glands of the bovine uterus, to activate the specific inflammatory cascade. In fact, this kind of weak and transient inflammation is required to remove excess and dead sperm remaining in the uterine lumen and to complete this clearance within several hours without tissue damage for providing the ideal uterine environment for acceptance of early embryo and implantation.

Quite recently, Kar et al. (40) predicted the 3-D structure of bovine TLR2 as a reliable computational tool. However, the structures of bovine TLR1 and TLR6 are unavailable. Hence, in the present study, the binding affinity of TLR2 with TLR1 and TLR6 was evaluated using known crystal structures of both mouse and human. The *in-silico* models showed the evidence for the stabilized interactions between TLR2/1 and TLR2/6 heterodimers in presence of their agonists. Notably, the present study provided the first insight for bovine TLR2 heterodimerization at molecular level. Furthermore, the data revealed that the main binding sites of bovine TLRs were identical to human and mouse and occupied by their specific ligands. Therefore, in complementary with the experimental investigations, it could be concluded that TLR2/1 dimerization occurs in bovine uterus in the presence of sperm. However, the sperm surface molecule/(s) which could initiate and/or regulate this TLR2/1 dimerization process for the specific inflammatory cascade remained to be investigated.

## Conclusion

**Figure 7:**
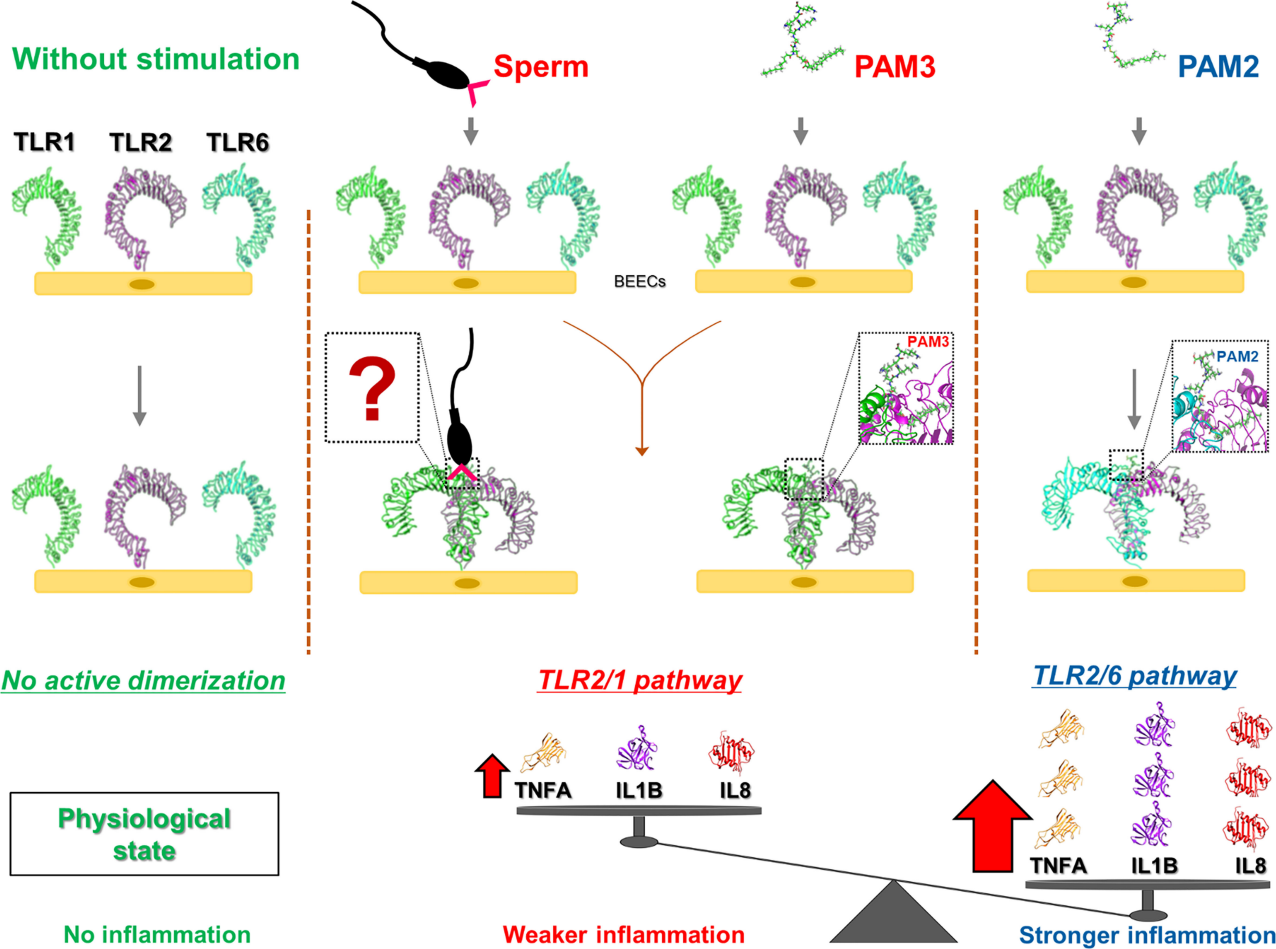
A graphic demo of our working hypothesis on the activation of possible TLR2 pathway signaling in sperm-induced inflammation in bovine endometrial epithelia. The model demonstrating that the stimulation of endometrial epithelia with different TLR2 agonist (PAM3: TLR2/1 and PAM2: TLR2/6) results in different intensity in inflammation response. In fact, initiating TLR2/1 signaling cascade could lead to weaker inflammation compared to TLR2/6. As for sperm-endometrial epithelial interaction, sperm induce TLR2/1 heterodimerization signaling, to trigger the weak and acute inflammation. We suppose that sperm may use small molecules from its surface (such as lipopeptides or glycans) to enhance TLR2/1 dimerization. However, further investigations are required to define the specific ligand(s) on the sperm cell membrane that regulate the initiation of TLR2/1 heterodimerization. “?” shows the unknown molecules form sperm surface side which may link TLR1 to TLR2.

Our results revealed that sperm utilize TLR2/1 pathway to induce a weak inflammatory response in bovine endometrium. Activation of TLR2/6 heterodimer could lead to an excessive inflammatory response compared to that of sperm or TLR2/1 (**Figure 7**). Further, *in-silico* findings revealed that the ectodomains of bovine TLR2 formed a hetero-dimer upon ligand binding to initiate cell signaling pathway. Altogether, our data strongly suggested that sperm utilize TLR2/1, but not TLR2/6, heterodimerization to induce the weak physiological inflammatory responses in the bovine uterus. However, further investigations are required to define the specific ligand(s) on the sperm cell membrane that is required to bridge and stabilize the TLR2/1 heterodimer.

## Data availability statement

The original contributions presented in the study are included in the article/**Supplementary Material**. Further inquiries can be directed to the corresponding author.

## Ethics statement

The animal study was reviewed and approved by Committee on the Ethics of Animal Experiments of the Obihiro University of Agriculture and Veterinary Medicine, Japan (Permit number 27-74).

## Author contributions

AlM, MY, RK, IA and AM conceived and designed the experiments. AlM, MY, NU and IA performed the experiments. AlM, MY and IA analyzed the data. AlM, RK and, AM provided reagents/materials/analysis tools. AlM, MY, RK, IA and AM wrote the manuscript. All authors contributed to the article and approved the submitted version.

## References

[1] Akthar I Suarez SS Morillo VA Sasaki M Ezz MA Takahashi KI. Sperm enter glands of preovulatory bovine endometrial explants and initiate inflammation. Reproduction (2020) 159:181–92. doi: 10.1530/REP-19-0414 31794421

[2] Ezz MA Marey MA Elweza AE Kawai T Heppelmann M Pfarrer C. TLR2/4 signaling pathway mediates sperm-induced inflammation in bovine endometrial epithelial cells *in vitro* . PloS One (2019) 14:1–17. doi: 10.1371/journal.pone.0214516 PMC6469758 30995239

[3] Marey MA Aboul Ezz M Akthar I Yousef MS Imakawa K Shimada M. Sensing sperm *via* maternal immune system: a potential mechanism for controlling microenvironment for fertility in the cow. J Anim Sci (2020) 98:S88–95. doi: 10.1093/jas/skaa147 PMC7433911 32810249

[4] Kowsar R Keshtegar B Miyamoto A. Understanding the hidden relations between pro-and anti-inflammatory cytokine genes in bovine oviduct epithelium using a multilayer response surface method. Sci Rep (2019) 9:1–17. doi: 10.1038/s41598-019-39081-w 30816156 PMC6395797

[5] Morillo VA Akthar I Fiorenza MF Takahashi K-I Sasaki M Marey MA. Toll-like receptor 2 mediates the immune response of the bovine oviductal ampulla to sperm binding. Mol Reprod Dev (2020) 87:1059–69. doi: 10.1002/mrd.23422 32914493

[6] Zinnah MA Marey MA Akhtar I Elesh IF Matsuno Y Elweza AE. Peptidoglycan disrupts early embryo-maternal crosstalk *via* suppression of ISGs expression induced by interferon-tau in the bovine endometrium. Biochem Biophys Res Commun (2020) 532:101–7. doi: 10.1016/j.bbrc.2020.08.006 32828539

[7] Elesh IF Marey MA Zinnah MA Akthar I Kawai T Naim F. Peptidoglycan switches off the TLR2-mediated sperm recognition and triggers sperm localization in the bovine endometrium. Front Immunol (2021) 11:619408. doi: 10.3389/fimmu.2020.619408 33643300 PMC7905083

[8] Elweza AE Ezz MA Acosta TJ Talukder AK Shimizu T Hayakawa H. A proinflammatory response of bovine endometrial epithelial cells to active sperm *in vitro* . Mol Reprod Dev (2018) 85:215–26. doi: 10.1002/mrd.22955 29337420

[9] Abdulrahman Alrabiah N Simintiras CA Evans ACO Lonergan P Fair T. Biochemical alterations in the follicular fluid of bovine peri-ovulatory follicles and their association with final oocyte maturation. Reprod Fertil (2022) 4:e220090. doi: 10.1530/raf-22-0090 36547396 PMC9874974

[10] Shimada M Yanai Y Okazaki T Noma N Kawashima I Mori T. Hyaluronan fragments generated by sperm-secreted hyaluronidase stimulate cytokine/chemokine production *via* the TLR 2 and TLR4 pathway in cumulus cells of ovulated COCs, which may enhance fertilization. Development (2008) 135:2001–11. doi: 10.1242/dev.020461 18434414

[11] Shimada M Hernandez-Gonzalez I Gonzalez-Robanya I Richards JAS. Induced expression of pattern recognition receptors in cumulus oocyte complexes: novel evidence for innate immune-like functions during ovulation. Mol Endocrinol (2006) 20:3228–39. doi: 10.1210/me.2006-0194 16931571

[12] Akthar I Marey MA Kim Y Shimada M Suarez SS Miyamoto A. Sperm interaction with the uterine innate immune system: toll-like receptor 2 (TLR2) is a main sensor in cattle. Reprod Fertil Dev (2021) 34:139–48. doi: 10.1071/RD21265 35231265

[13] Kang JY Nan X Jin MS Youn SJ Ryu YH Mah S. Recognition of lipopeptide patterns by toll-like receptor 2-toll-like receptor 6 heterodimer. Immunity (2009) 31:873–84. doi: 10.1016/j.immuni.2009.09.018 19931471

[14] Jin MS Kim SE Heo JY Lee ME Kim HM Paik SG. Crystal structure of the TLR1-TLR2 heterodimer induced by binding of a tri-acylated lipopeptide. Cell (2007) 130:1071–82. doi: 10.1016/j.cell.2007.09.008 17889651

[15] Li JB Lee DSW Madrenas J. Evolving bacterial envelopes and plasticity of TLR2-dependent responses: basic research and translational opportunities. Front Immunol (2013) 4:347. doi: 10.3389/fimmu.2013.00347 24191155 PMC3808894

[16] DePaolo RW Tang F Kim IY Han M Levin N Ciletti N. Toll-like receptor 6 drives differentiation of tolerogenic dendritic cells and contributes to LcrV-mediated plague pathogenesis. Cell Host Microbe (2008) 4:350–61. doi: 10.1016/j.chom.2008.09.004 PMC2633104 18854239

[17] Jones S Thornton JM. Principles of protein-protein interactions. Proc Natl Acad Sci U.S.A. (1996) 93:13–20. doi: 10.1073/pnas.93.1.13 8552589 PMC40170

[18] Mansouri A Kowsar R Zakariazadeh M Hakimi H Miyamoto A. The impact of calcitriol and estradiol on the SARS-CoV-2 biological activity: a molecular modeling approach. Sci Rep (2022) 12:1–15. doi: 10.1038/s41598-022-04778-y 35027633 PMC8758694

[19] Su L Wang Y Wang J Mifune Y Morin MD Jones BT. Structural basis of TLR2/TLR1 activation by the synthetic agonist diprovocim. J Med Chem (2019) 62:2938–49. doi: 10.1021/acs.jmedchem.8b01583 PMC6537610 30829478

[20] Bouzari S Savar N. In silico study of ligand binding site of toll-like receptor 5. Adv BioMed Res (2014) 3:41. doi: 10.4103/2277-9175.125730 24627849 PMC3949343

[21] Basith S Manavalan B Govindaraj RG Choi S. In silico approach to inhibition of signaling pathways of toll-like receptors 2 and 4 by ST2L. PloS One (2011) 6:e23989. doi: 10.1371/journal.pone.0023989 21897866 PMC3163686

[22] van Zundert GCP Rodrigues JPGLM Trellet M Schmitz C Kastritis PL Karaca E. The HADDOCK2.2 web server: user-friendly integrative modeling of biomolecular complexes. J Mol Biol (2016) 428:720–5. doi: 10.1016/j.jmb.2015.09.014 26410586

[23] Wallace AC Laskowski RA Thornton JM. LIGPLOT: a program to generate schematic diagrams of protein-ligand interactions. Protein Eng (1995) 8:127–34. doi: 10.1093/protein/8.2.127 7630882

[24] Lee J Cheng X Swails JM Yeom MS Eastman PK Lemkul JA. CHARMM-GUI input generator for NAMD, GROMACS, AMBER, OpenMM, and CHARMM/OpenMM simulations using the CHARMM36 additive force field. J Chem Theory Comput (2016) 12:405–13. doi: 10.1021/acs.jctc.5b00935 PMC4712441 26631602

[25] Abraham MJ Murtola T Schulz R Páll S Smith JC Hess B. GROMACS: high performance molecular simulations through multi-level parallelism from laptops to supercomputers. SoftwareX (2015) 1–2:19–25. doi: 10.1016/j.softx.2015.06.001

[26] Brooks BR Brooks CL 3rd Mackerell AD Jr Nilsson L Petrella RJ Roux B. CHARMM: the biomolecular simulation program. J Comput Chem (2009) 30:1545–614. doi: 10.1002/jcc.21287 PMC2810661 19444816

[27] Yang J Yan R Roy A Xu D Poisson J Zhang Y. The I-TASSER suite: protein structure and function prediction. Nat Methods (2015) 12:7–8. doi: 10.1038/nmeth.3213 PMC4428668 25549265

[28] Kumari R Kumar R Lynn A. G_mmpbsa–a GROMACS tool for high-throughput MM-PBSA calculations. J Chem Inf Model (2014) 54:1951–62. doi: 10.1021/ci500020m 24850022

[29] Farrokhpour H Mansouri A Najafi Chermahini A. Transport behavior of the enantiomers of lactic acid through the cyclic peptide nanotube: enantiomer discrimination. J Phys Chem C (2017) 121:8165–76. doi: 10.1021/acs.jpcc.7b00010

[30] Farrokhpour H Mansouri A Rajabi AR Najafi Chermahini A. The effect of the diameter of cyclic peptide nanotube on its chirality discrimination. J Biomol Struct Dyn (2019) 37:691–701. doi: 10.1080/07391102.2018.1436090 29393002

[31] Mansouri A Mahnam K. Designing new surfactant peptides for binding to carbon nanotubes *via* computational approaches. J Mol Graph Model (2017) 74:61–72. doi: 10.1016/j.jmgm.2017.02.016 28359959

[32] Barzegar A Mansouri A Azamat J. Molecular dynamics simulation of non-covalent single-walled carbon nanotube functionalization with surfactant peptides. J Mol Graph Model (2016) 64:75–84. doi: 10.1016/j.jmgm.2016.01.003 26811869

[33] Volkamer A Kuhn D Rippmann F Rarey M. DoGSiteScorer: a web server for automatic binding site prediction, analysis and druggability assessment. Bioinformatics (2012) 28:2074–5. doi: 10.1093/bioinformatics/bts310 22628523

[34] Fährrolfes R Bietz S Flachsenberg F Meyder A Nittinger E Otto T. ProteinsPlus: a web portal for structure analysis of macromolecules. Nucleic Acids Res (2017) 45:W337–43. doi: 10.1093/nar/gkx333 PMC5570178 28472372

[35] Sepehri Z Kiani Z Nasiri AA Kohan F. Toll-like receptor 2 and type 2 diabetes. Cell Mol Biol Lett (2016) 21:2. doi: 10.1186/s11658-016-0002-4 28536605 PMC5415836

[36] Chau TA McCully ML Brintnell W An G Kasper KJ Vinés ED. Toll-like receptor 2 ligands on the staphylococcal cell wall downregulate superantigen-induced T cell activation and prevent toxic shock syndrome. Nat Med (2009) 15:641–8. doi: 10.1038/nm.1965 19465927

[37] Schaefer TM Desouza K Fahey JV Beagley KW Wira CR. Toll-like receptor (TLR) expression and TLR-mediated cytokine/chemokine production by human uterine epithelial cells. Immunology (2004) 112:428–36. doi: 10.1111/j.1365-2567.2004.01898.x PMC1782499 15196211

[38] Murgueitio MS Henneke P Glossmann H Santos-Sierra S Wolber G. Prospective virtual screening in a sparse data scenario: design of small-molecule TLR2 antagonists. ChemMedChem (2014) 9:813–22. doi: 10.1002/cmdc.201300445 24470159

[39] Monlish DA Greenberg ZJ Bhatt ST Leonard KM Romine MP Dong Q. TLR2/6 signaling promotes the expansion of premalignant hematopoietic stem and progenitor cells in the NUP98–HOXD13 mouse model of MDS. Exp Hematol (2020) 88:42–55. doi: 10.1016/j.exphem.2020.07.001 32652111 PMC7673652

[40] Kar PP Araveti PB Kuriakose A Srivastava A. Design of a multi-epitope protein as a subunit vaccine against lumpy skin disease using an immunoinformatics approach. Sci Rep (2022) 12:1–11. doi: 10.1038/s41598-022-23272-z 36371522 PMC9653426

