# Supplementary material for: Sperm activate TLR2/TLR1 heterodimerization to induce a weak proinflammatory response in the bovine uterus
Source: Front Immunol. 2023 Apr 27;14:1158090. doi: 10.3389/fimmu.2023.1158090 (PMC10174305; doi:10.3389/fimmu.2023.1158090)
Supplement: Supplementary file 1 [file DataSheet_1.docx]

***Supplementary Material***

**Sperm activate TLR2/TLR1 heterodimerization to induce a weak proinflammatory response in bovine uterus**

**Alireza Mansouri, Mohamed Samy Yousef, Rasoul Kowsar, Nonoka Usui, Ihshan Akthar and Akio Miyamoto^*^**

***Corresponding author:**

Akio Miyamoto, Ph.D.

E-mail: [akiomiya@obihiro.ac.jp](mailto:akiomiya@obihiro.ac.jp)

# Supplementary Data

**MD simulation and MM/PBSA calculations**

All MD simulations for free molecules were carried out in four steps (minimization, NVT, NPT and MD production) of the box of water with adding Na^+^ and Cl^−^ ions to access 140 mM ionic strength. The entire systems were minimized initially by the steepest descent algorithm with 50000 cycles without any included position restrictions, followed by the equilibration process, a 100 ps NVT set of MD, then a 100 ps NPT set of restrictions of TLRs and ligands at the 1000 kJ/mol·nm^−2^ harmonic force constant in the NPT phase. In the production step, 100 ns (for optimizing all molecules) and 150 ns (for optimizing TLRs/ligands) MD simulations were performed with no included position restraints. The TIP3P water model was used in this study to design the solvation box of molecules with applying 1.5 nm for minimum distance between the solute and the box walls. The simulations were conducted at a temperature of 300 K with a time step of 2 fs, using LINCS algorithm and considering the periodic boundary condition (PBC) in equilibration and production processes, by GROMACS 2020 with CHARMM 27 force field parameters. SwissParam Web site was used to obtain whole topologies of ligands based on the CHARMM force field parameters. CHARMM-GUI was employed to model N-glycan of TLRs (as shown in **Supplementary Scheme**). Parameters that were analyzed include RMSD (Root mean squared deviation), the radius of gyration, and RMSF (Root mean squared fluctuations) as a time-dependent function of MDS (**Supplementary Figure 1-3**).

In this study, MM/PBSA method was selected to calculate the binding free energy between molecules from the MD production trajectories at the last 10 ns. MM/PBSA calculate electrostatic, van der Waals, polar solvation, solvent accessible surface area (SASA), solvent accessible volume (SAV), Weeks−Chandler−Andersen (WCA) energies. The binding free energy is the summation of the aforementioned energies. In order to confirm and validate the results obtained through MD simulation and MM/PBSA, definite experimental data (KD) from prior research were compared to our calculated binding free energy, and quantitative correlations were investigated between these two parameters (KD and binding free energy). To aim that, ChemSketch was used to generate the two-dimensional (2-D) structures of TLR2 antagonists (five compounds) and then the three-dimensional (3-D) structures were created using PRODRG2 server. 100 ns MD simulations time were finally conducted to optimize the antagonist molecules. Afterward, the compounds with KD experimental data (extracted from an experimental research) were docked to the main binding site of TLR2, then binding free energy was considered using MD simulation and the MM/PBSA method. The results demonstrated a proper correlation coefficient (R^2^) between binding free energy (obtained by MM-PBSA) method and experimental KD data (R^2^ = 0.77). The result and final structures were depicted in **Supplementary Figure 4**.

# Supplementary results

**The reach of the equilibrium in the simulation of TLRs**

**Supplementary Figure 1** indicated the root mean square deviations (RMSDs) of TLR protein backbone in all used complexes during MD simulation. The RMSDs value of the TLRs were oscillating between 0.2–0.4 nm over the simulation time (150 ns), which confirmed reaching the equilibrium state from the starting point.

**Calculated affinity between TLRs in dimer forms in humans and mouse models**

There was no binding affinity between TLR2/2 as a homodimer and TLR2/1 as a heterodimer form **(Supplementary Table 3)**; the average energies were 124.9 for TLR2/1. Several hydrogen bonds and hydrophobic interactions between residues were identified for heterodimer forms **(Supplementary Figure 6A and Supplementary Figure 8)**. Concerning TLR2/6 heterodimer, there was a binding affinity between TLR2 and TLR6 with average energies -176.47 Kcal/mol. However, such energy is not enough to stabilize those macromolecules beside each other. Furthermore, the contribution of Van der Waals (vdW) interaction in TLR2/6 (-92.6 *±* 10.6 Kcal/mol) was relatively higher than TLR2/1 (-65.44 *±* 5.8 Kcal/mol).

**The impact of agonists on TLRs dimerization**

The binding free energies (BFE) obtained by MM/PBSA for agonists to the main binding site of TLRs were demonstrated in **Supplementary Table 4**. The MM/PBSA result emphasized that the interaction of agonists with TLR2 is much stronger than TLR1 and TLR6. For instance, the BFE between TLR2-PAM3 was -57 Kcal/mol, however as for TLR1-PAM3 showed a higher BFE (-22.8 Kcal/mol). Concerning TLR2-PAM2, the BFE was -64.6, however regarding TLR6-PAM2 the BFE increased to -5.6 Kcal/mol. The residues involved in the interaction with agonists were indicated in **Supplementary Figure 6B**. Agonists showed significant binding affinities to the main binding sites of TLRs that could play a critical role in bridging between TLRs. PAM3 showed a potent impact compared to PAM2, owing to have an Amide bound lipid.

With regards to the protein folding of TLRs in the absence and presence of agonists, in all investigated TLRs heterodimers, the radius of gyration showed a slight shift in particular during the last 50 ns in absence of their agonists, i.e., compactness of TLR2 heterodimer is due to a presence of bridging ligand (**Supplementary Figure 2**). However, the residual flexibility of TLRs complexes, measured by the root means square fluctuation (RMSF), was almost identical in the presence or absence of agonists throughout the simulation time (**Supplementary Figure 3**).

**Supplementary Scheme.** The sequence of glycans attached to amino acid side chain (Asparagine residue) in TLRs glycoproteins. aDGlcNAc: α-N-acetyl-D-glucosamine, bDGlcNAc: β-N-acetyl-D-glucosamine, DMan: D-mannose.

**TLR2/1 complex (PDB code: 2Z7X)**

**TLR2:**

aDGlcNAc(1→)Asn199

bDGlcNAc(1→)Asn414

bDGlcNAc(1→)Asn442

**TLR1:**

aDMan(1→4)aDGlcNAc(1→4)bDGlcNAc(1→)Asn51

aDMan(1→4)bDGlcNAc(1→4)bDGlcNAc(1→)Asn330

bDMan(1→4)bDGlcNAc(1→4)bDGlcNAc(1→)Asn429

bDGlcNAc(1→)Asn163

**TLR2/6 complex (PDB code: 3A79)**

**TLR2:**

bDGlcNAc(1→)Asn147

bDGlcNAc(1→)Asn414

bDGlcNAc(1→4)bDGlcNAc(1→)Asn442

**TLR6:**

bDGlcNAc(1→4)bDGlcNAc(1→)Asn253

bDMan(1→4)bDGlcNAc(1→4)bDGlcNAc(1→)Asn285

bDMan(1→4)bDGlcNAc(1→4)bDGlcNAc(1→)Asn359

bDMan(1→4)bDGlcNAc(1→4)bDGlcNAc(1→)Asn401

bDGlcNAc(1→4)aDGlcNAc(1→)Asn434

bDGlcNAc(1→)Asn144

bDGlcNAc(1→)Asn214

bDGlcNAc(1→)Asn195

# Supplementary Figures and Tables

## Supplementary Figures


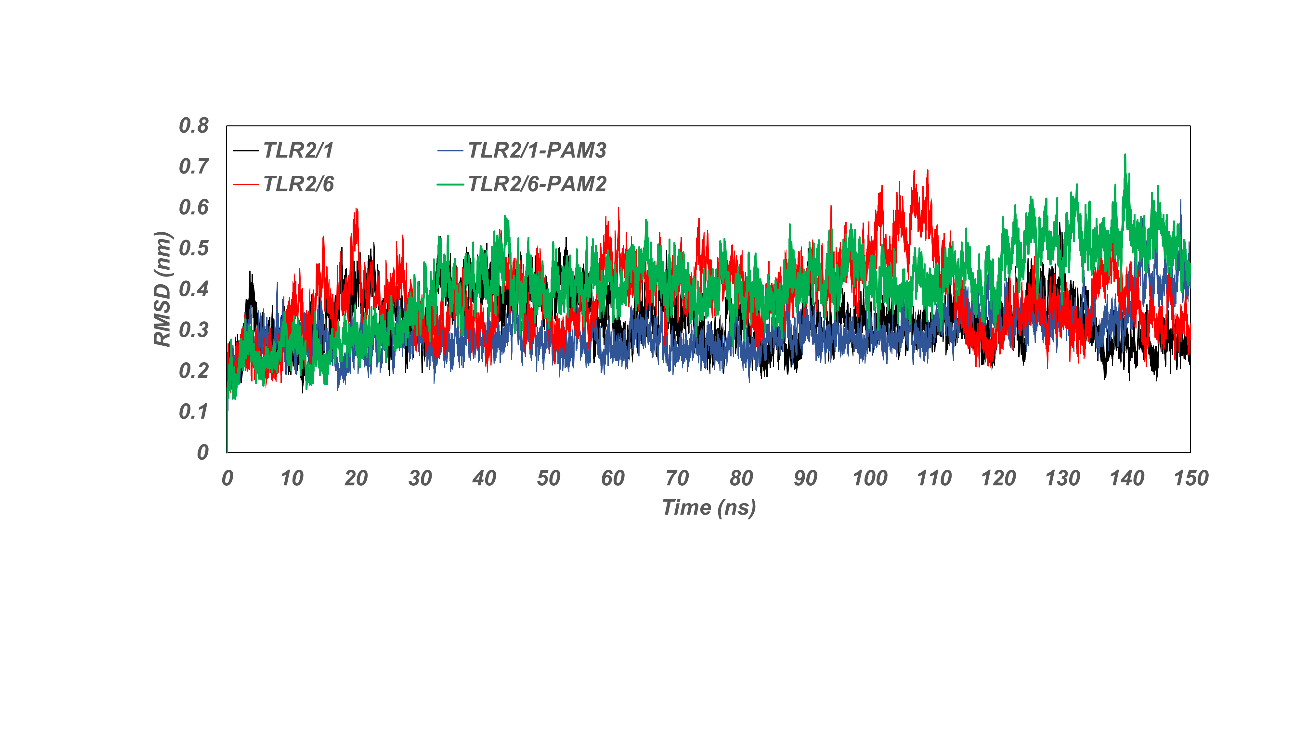


**Supplementary Figure 1.** Root mean square deviations (RMSD) of TLRs backbone during 150 ns MD simulation times


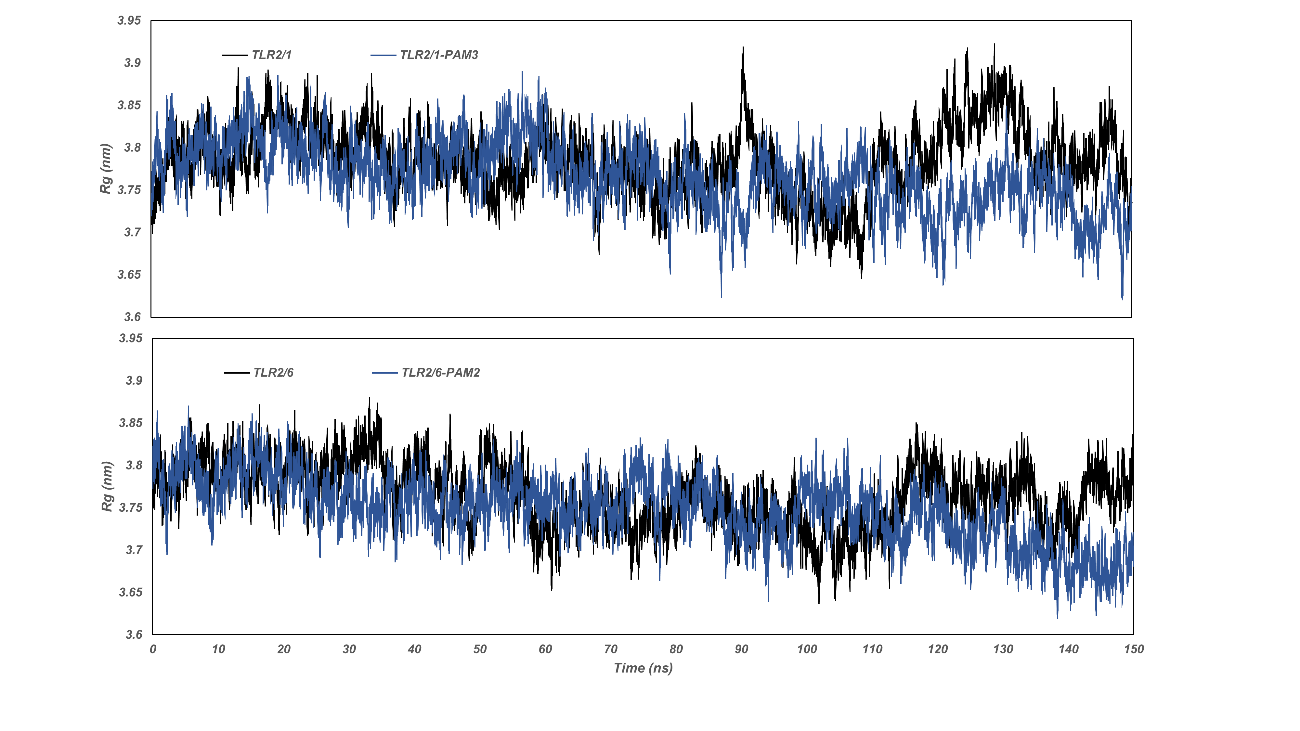


**Supplementary Figure 2.** The gyration radius (Rg) of TLRs dimer in the absence and presence of agonists (PAM3 or PAM2) following 150 ns MD simulation.


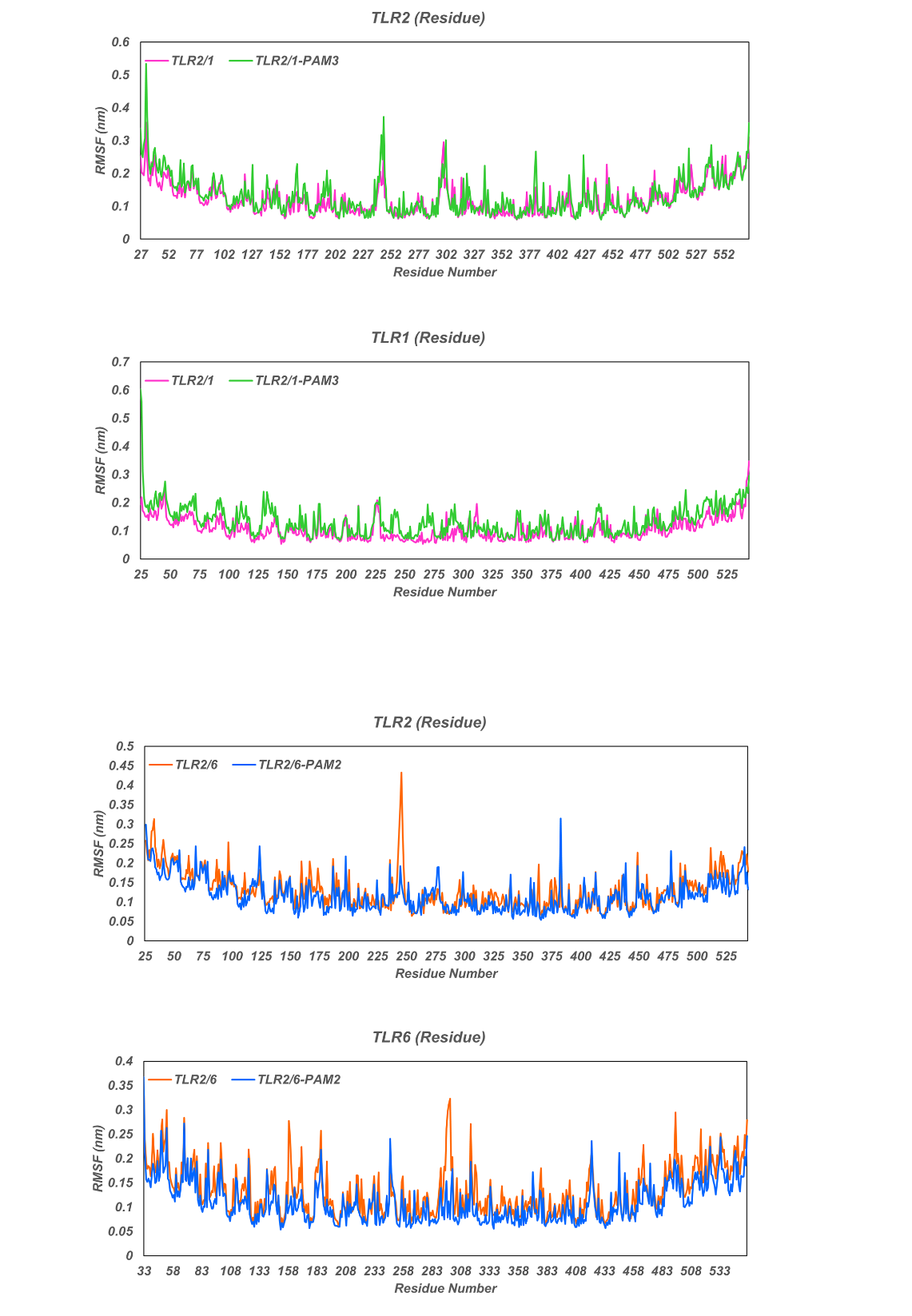


**Supplementary Figure 3.** The Root mean square fluctuation (RMSF) each residue in TLRs dimer systems in the absence and presence of agonist (PAM3 or PAM2).


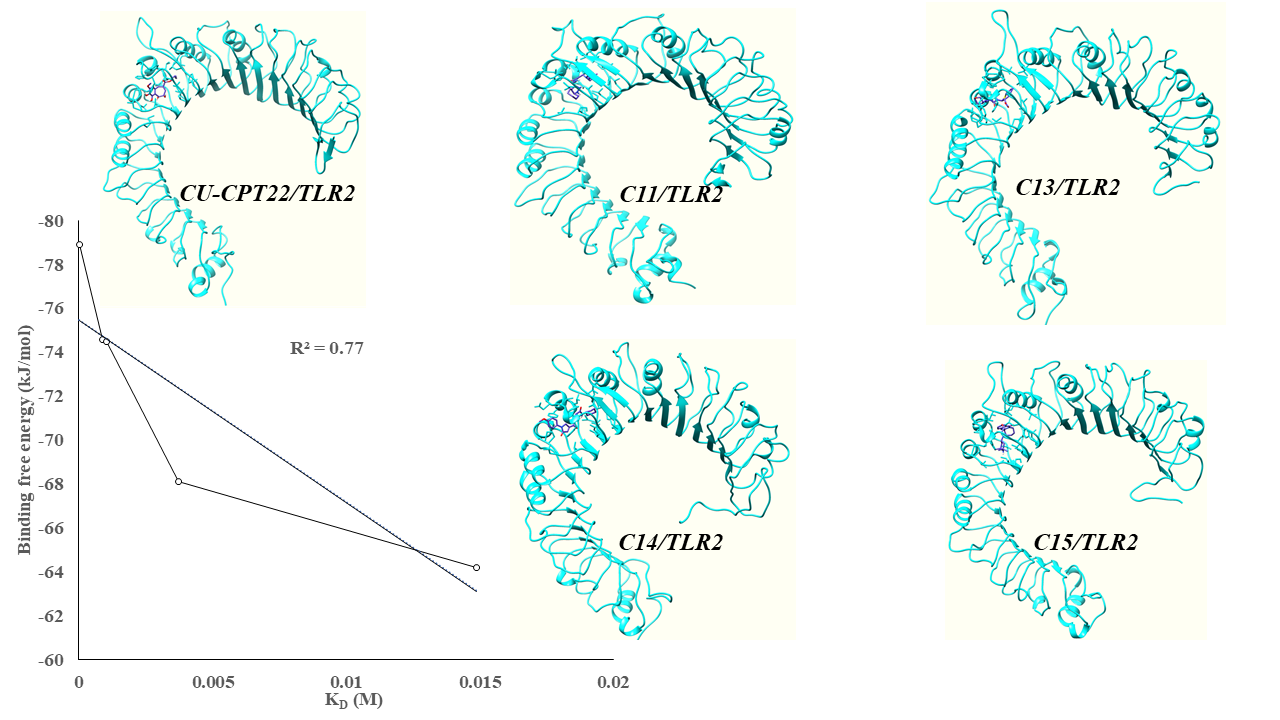


**Supplementary Figure 4.** The correlation coefficient (R^2^) between the calculated binding free energy (MM/PBSA) and experimental K_D_ data. The K_D_ value for CU-CPT22 (known TLR2 antagonist), Compound 11 (C11), Compound 13 (C13), Compound 14 (C14) and Compound 15 (C15) is 0.00000583, 0.00087, 0.00372, 0.00102 and 0.0149, respectively. R^2^ between binding free energy and K_D_.


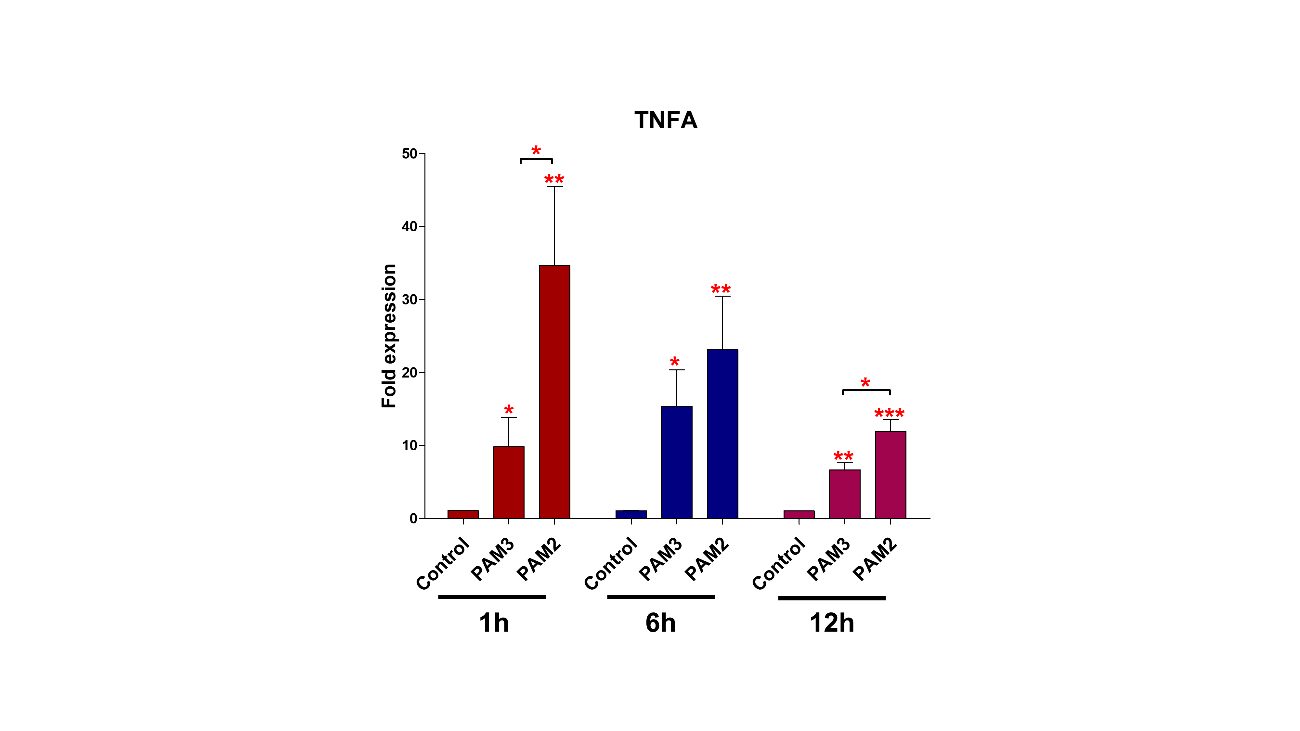


**Supplementary Figure 5.** Relative mRNA expression of major inflammatory cytokines at 1, 6 and 12 h after stimulation. The PAM2 treatment group lowers pro-inflammatory gene expression with time. Asterisks show a considerable variance (*P < 0.05, **P < 0.01, or ***P < 0.001) between the treatment groups.


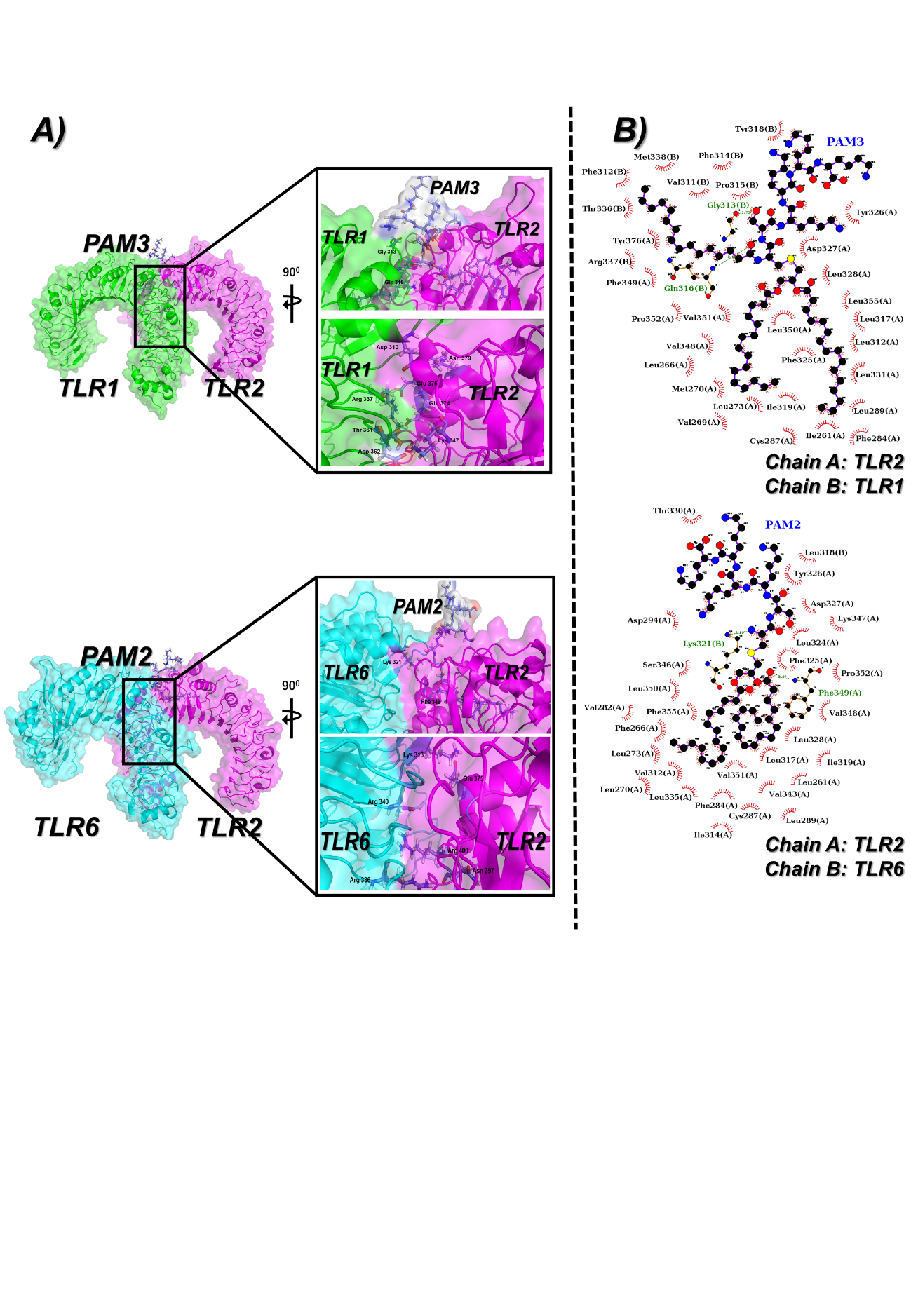


**Supplementary Figure 6.** Analyzing the final structure of TLR2/1-PAM3, TLR2/6-PAM2 and TLR2/2-Diprovocim complexes after applying 150 ns MD simulation. **A)** the final 3-D structure TLRs heterodimer and homodimer in presence of agonists, obtained from trajectory MD simulation. TLR1, TLR2 and TLR6 colored by Green, Magenta and Cyan, respectively; agonists indicated by stick. **B)** 2-D agonists-TLRs interaction diagrams obtained through LIGPLOT software. The figure is showing whole residues of TLRs which interact with agonists to stabilize TLR2 heterodimer or homodimer. The spoked arcs illustrate TLRs (chain A and B) making non-covalent contacts with agonists.


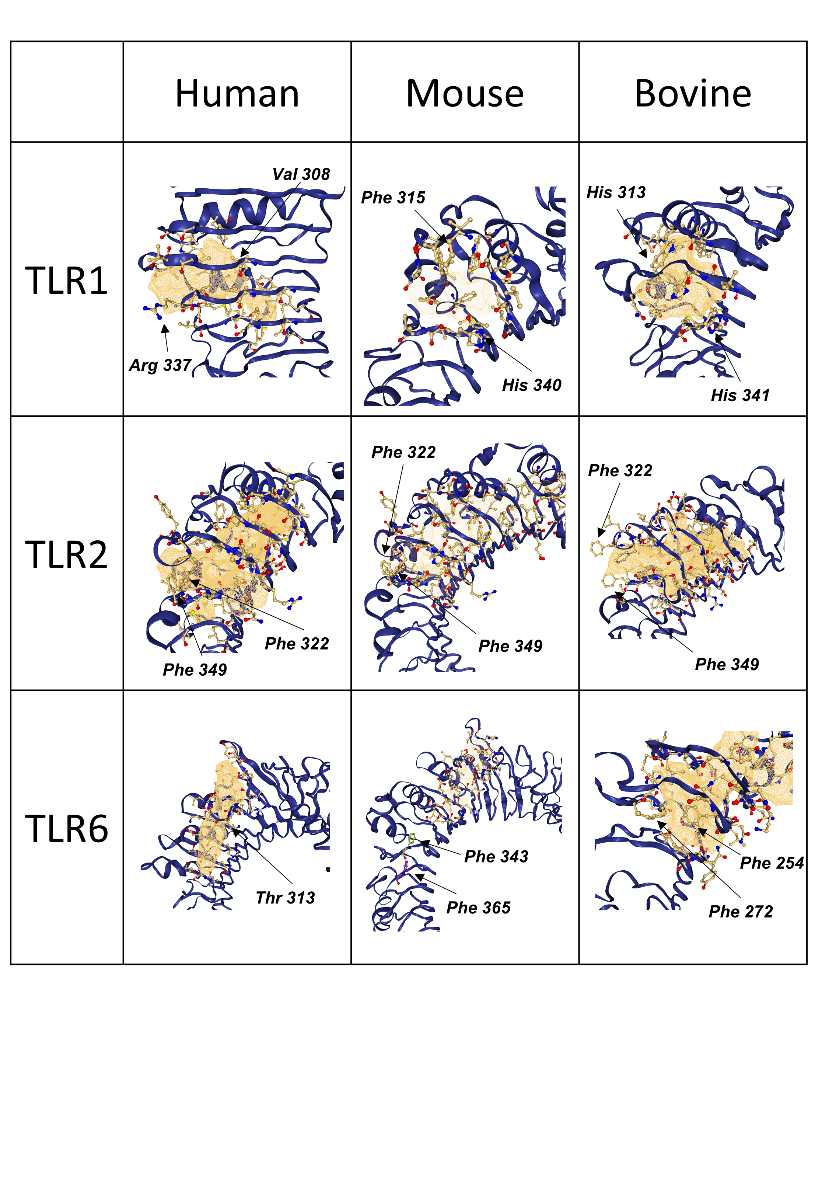


**Supplementary Figure 7.** The first predicted binding site of TLRs in three species by DoGSite Scorer.


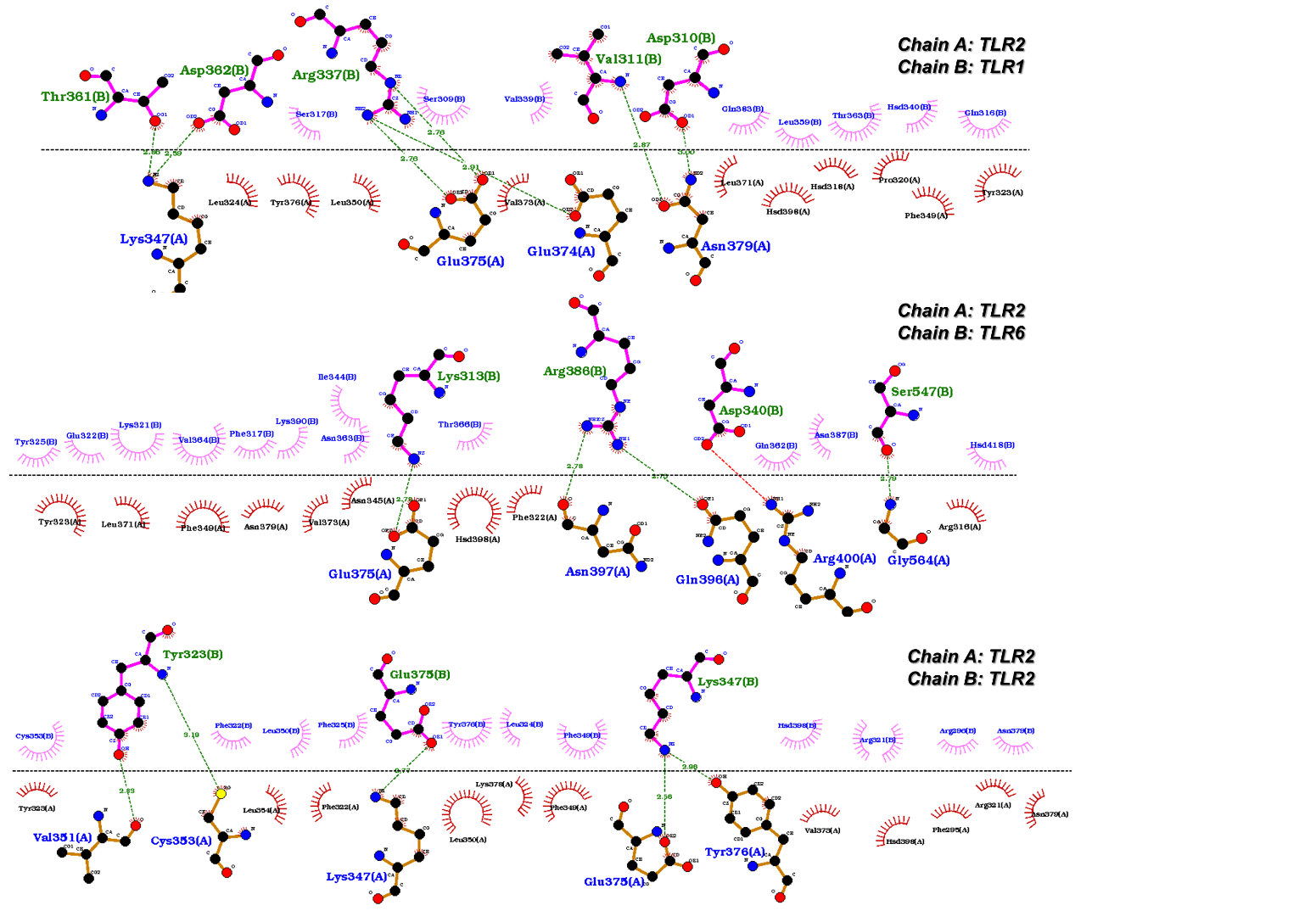


**Supplementary Figure 8.** 2-D TLR2-TLR1, TLR2-TLR6 and TLR2-TLR2 interaction diagrams obtained through Ligplot software. This figure is indicating whole residues in the interface of TLR2 heterodimer and homodimer. The spoked arcs illustrate TLR2 (chain A) making non-covalent contacts with TLR1, TLR2 and TLR6 (chain B).

## Supplementary Tables

**Supplementary Table 1.** List of primers used in real-time PCR

| Gene | Primer | Sequence of nucleotide (5′⇒3′) | Accession No. |
| --- | --- | --- | --- |
| β-actin | F  R | TCACCAACTGGGACGACATG  CGTTGTAGAAGGTGTGGTGCC | AY141970.1 |
| TNFA | F  R | CAAAAGCATGATCCGGGATG  TTCTCGGAGAGCACCTCCTC | NM_173966.3 |
| IL1B | F  R | AATCGAAGAAAGGCCCGTCT  ATATCCTGGCCACCTCGAAA | NM_174093.1 |
| IL8 | F  R | CCAATGGAAACGAGGTCTGC  CCTTCTGCACCCACTTTTCCT | NM_173925.2 |
| PGES1 | F  R | AAAATGTACGTGGTGGCCGT  CTTCTTCCGCAGCCTCACTT | NM_174443.2 |
| TLR2 | F  R | CATGGGTCTGGGCTGTCATC  CCTGGTCAGAGGCTCCTTCC | NM_174197.2 |
| TLR1 | F  R | ACCCTACTCTGAACCTCAAG  GACTGCACACTGGATTTCTG | NM_001046504.1 |
| TLR6 | F  R | CTCCGGGAGATAGTCACTTC  GGCCCTGGATTCTATTATGG | NM_001001159.1 |

**Supplementary Table 2.** List of antibodies used for immunofluorescence (IF)

**Supplementary Table 3.** Binding free energy (Kcal/mol) calculated by MM/PBSA methods for TLRs dimers during the last 10 ns of MD simulation.

| **PPI** | **Crystal structure (with agonist)** | **Crystal structure (without agonists)** | **Initial structure**  **(by Haddock)** | **Average** | **Standard deviation** |
| --- | --- | --- | --- | --- | --- |
| **TLR2-TLR1** | 104.25 | 168.8 | 101.91 | 124.9 | 37.9 |
| **TLR2-TLR6** | -167.04 | -198.97 | -163.4 | -176.47 | 15.9 |

**Supplementary Table 4.** The result of binding free energy (Kcal/mol) calculated by MM/PBSA methods for TLR/agonist complexes during the last 10 ns of MD simulation

|  | PAM3 | PAM2 |
| --- | --- | --- |
| TLR2 | -57 | -64.6 |
| TLR1 | -22.8 | NA |
| TLR6 | NA | -5.6 |

**Supplementary Table 5.** The physicochemical properties (volume, surface and drugScore) of the first predicted binding site by DoGSite Scorer.

|  |  | **volume (A^3^)** | **surface (A^2^)** | **drugScore** |
| --- | --- | --- | --- | --- |
| **TLR1** | **Human** | 533.93 | 538.7 | 0.8 |
|  | **Mouse** | 370.52 | 380.74 | 0.7 |
|  | **Bovine** | 500.76 | 498.58 | 0.7 |
| **TLR2** | **Human** | 1803.89 | 1867.19 | 0.8 |
|  | **Mouse** | 1490.3 | 1170.56 | 0.8 |
|  | **Bovine** | 1418.18 | 1434.77 | 0.8 |
| **TLR6** | **Human** | 496.1 | 692.31 | 0.7 |
|  | **Mouse** | 460.56 | 653.29 | 0.7 |
|  | **Bovine** | 1335.24 | 1671.48 | 0.8 |
